# Correlated shapeshifting and configurational isomerization†

**DOI:** 10.1039/d4sc03699a

**Published:** 2024-08-23

**Authors:** Burhan A. Hussein, William Maturi, Mary Kate Rylands, Aisha N. Bismillah, Yuzhen Wen, Juan A. Aguilar, Rabia Ayub, Conor D. Rankine, Paul R. McGonigal

**Affiliations:** a Department of Chemistry, Durham University, Lower Mountjoy Stockton Road Durham DH1 3LE UK paul.mcgonigal@york.ac.uk; b Department of Chemistry, University of York Heslington York YO10 5DD UK

## Abstract

Herein we demonstrate that the rapid ‘shapeshifting’ constitutional isomerization of a substituted bullvalene is influenced by the *E*-to-*Z* configurational isomerization of a remote carbamate group, giving rise to correlated motion. We find that, while the *E*-configurational isomer of a bulky carbamate favors the *β*-bullvalene constitutional isomer, a noncovalent bonding interaction within the *Z*-carbamate tips the equilibrium toward the *γ*-bullvalene form. Using DFT modelling and NMR spectroscopy, this long-range interaction is identified as being between the bullvalene core and a pendant phenyl group connected to the carbamate. Coupling the constitutional changes of a bullvalene to a reciprocal configurational isomerization through a long-range interaction in this way will allow shapeshifting rearrangements to be exploited as part of collective motion in extended structures.

## Abstract 2

Rearrangements of a bullvalene cage between constitutional isomers are directed by configurational isomerization of a pendant group through noncovalent interactions.

## Graphical abstract

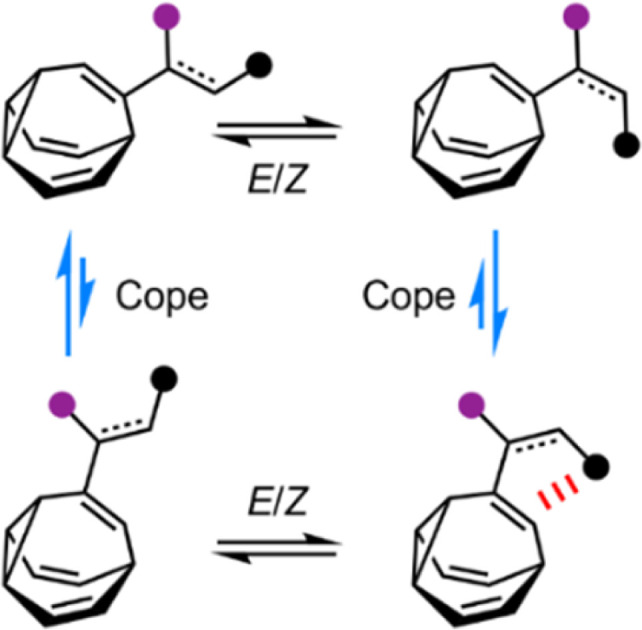

## Introduction

**Fig. 1:**
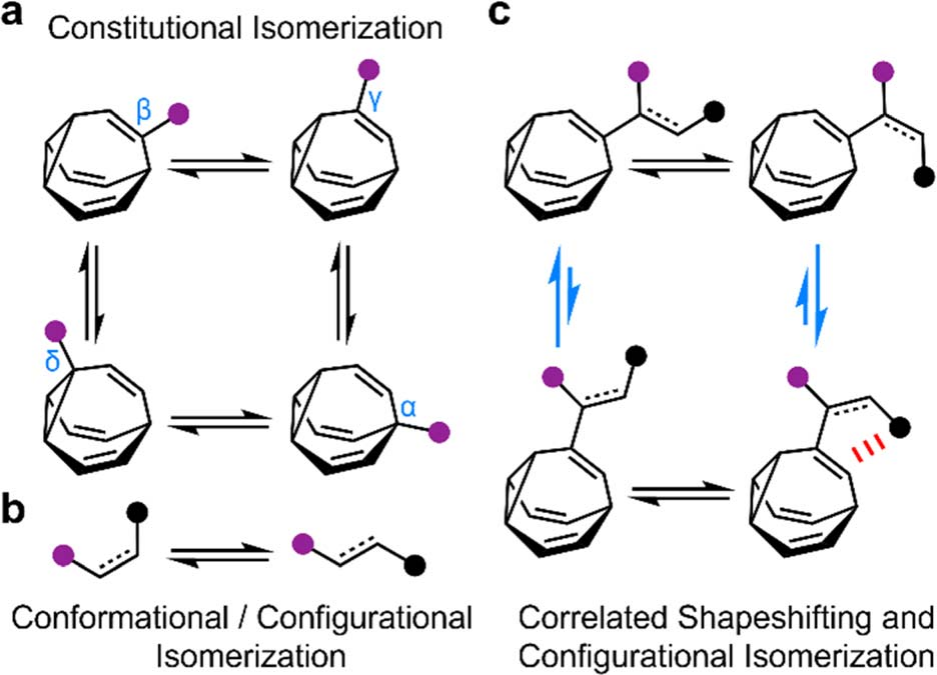
The combination of (a) bullvalene shapeshifting rearrangements between constitutional isomers with (b) conformational and configurational changes by single bond rotations and *E*-to-*Z* isomerization are (c) correlated through noncovalent interactions (red) to bias the two bullvalene rearrangement equilibria in opposite directions (blue arrows). Black and purple circles represent appended functional groups.

The rapid, successive Cope rearrangements of bullvalenes^1^ produce a series (Fig. 1a) of constitutional isomers. Recent advances in the synthesis of substituted derivatives,^2^ alongside the development of related fluxional cages,^3^ have led to renewed interest in exploiting these ‘shapeshifting’ structures as part of functional molecules and materials. Part of the appeal of using bullvalene derivatives is that they juxtapose the rigidity and well-defined bond angles^1*e*^ that are typical of cage-like structures with the rapid structural dynamics that are commonly only found in flexible molecules. They exhibit ‘rigid dynamics’ at the single-molecule level.^4^ Accordingly, investigations have been reported into bullvalene-containing fluorophores,^5^ antibiotics,^6^ small-molecule receptors,^7^ transition metal complexes,^8^ rigid-rod polymers,^9^ and single-molecule junctions.^10^

By contrast, most organic structures, particularly those containing rotatable single bonds, rapidly sample stereoisomeric structures through changes in bond angles and dihedral angles.^11^ The resulting isomerization (Fig. 1b) is not only influenced by local electronic and steric factors, but also by long-range interactions. Bond rotations of groups that are separated by several covalent bonds but are close together in space can, therefore, become correlated with one another,^12^*i.e.*, the isomerization or motion of one part of the molecule is coupled to a reciprocal conformational or configurational change in another part.

**Scheme 1:**
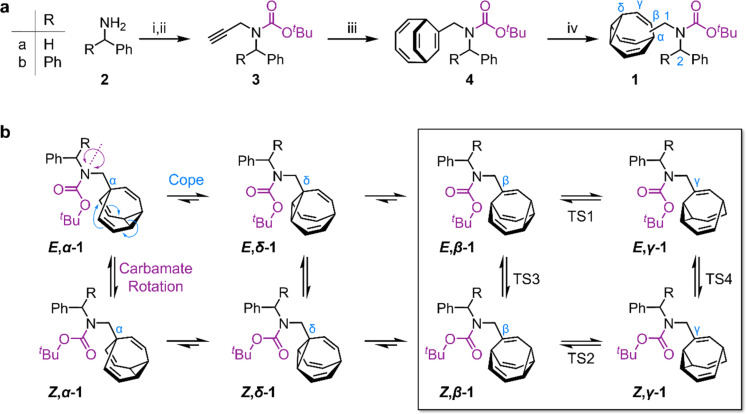
(a) Synthesis of sterically crowded bullvalene carbamates. Reagents and conditions: (i) 2, Boc_2_O, NEt_3_, CH_2_Cl_2_, rt, 24 h; (ii) 1, NaH, DMF, 0 °C, 30 min, 2. propargyl bromide, rt, 5 h, 70% 3a from 2a, 64% 3b from 2b; (iii) 3, cyclooctatetraene, CoBr_2_(dppe), ZnI_2_, Zn, TFE, 55 °C, 18 h, 55% 4a, 50% 4b; (iv) 4, *hν* (365 nm), thioxanthone, THF, 25 °C, 3 h, 50% 1a, 54% 1b. DMF = dimethylformamide, dppe = 1,2-bis(diphenylphosphino)ethane, TFE = 2,2,2-trifluoroethanol, and THF = tetrahydrofuran. (b) Eight isomers interchange by Cope rearrangements (horizontal equilibria) and by rotation around the carbamate N–C bond (vertical equilibria).

To exploit the shapeshifting rearrangements of bullvalenes in larger collective motions of extended structures,^12*d*–*f*^ it is necessary to understand how their fluxional Cope rearrangements are influenced (Fig. 1c) by isomerization of surrounding groups. Here, we report the isomeric distribution of carbamate-functionalized bullvalenes 1 (Scheme 1) and demonstrate that, even in this structurally simple case, conformational changes can become correlated to shapeshifting isomerization. For one of the compounds (benzhydryl derivative 1b), we find that while the *E*-carbamate is most stable as its *β*-substituted bullvalene isomer, bond rotation to the *Z*-carbamate biases the bullvalene unit towards its *γ*-substituted form instead, *i.e.*, the energetics of bullvalene isomerization and carbamate rotation are coupled to one another. We show that through-space interactions subtly alter the energetics of the dynamic system. As part of this investigation, we have also assessed the extent to which this isomer distribution can be accurately modelled by comparing the calculated energies of the lowest energy conformers, as opposed to analyzing the full conformational landscape.

## Results and discussion

We targeted (Scheme 1) tertiary carbamates 1a and 1b derived from benzylamine 2a and benzhydrylamine 2b, respectively. The partial double bond character of the carbamate C–N bond (*c.f.*, tertiary amide C–N bonds^13^) gives rise to *E*-to-*Z* configurational isomerization that can be slowed down and observed by dynamic NMR (DNMR) spectroscopy at low temperatures, which we reasoned would allow us to experimentally measure distributions of the configurational and constitutional isomers of 1a and 1b.

Starting from 2, we performed (Scheme 1a) a sequence of carbamate formation, alkylation with propargyl bromide, and cobalt(i)-catalyzed [6 + 2] cycloaddition with cyclooctatetraene^2*c*,14^ to produce bicyclo[4.2.2]deca-2,4,7,9-tetraene intermediates 4. A final photochemical di-π-methane rearrangement step^15^ using thioxanthone^2*e*^ as a photosensitizer gave rise to the target bullvalenes 1.

There are four possible constitutional isomers of 1 (Scheme 1b), which we label as *α*–*δ* according to the attachment point of the carbamate substituent to the bullvalene cage.^1*e*^ Consecutive Cope rearrangements from the *α*-isomer lead to the *δ*-, *β*-, then *γ*-isomers in a reversible linear sequence. Alternatively, rotation around the carbamate C–N bond interconverts the *E*- and *Z*-configurational isomers. In combination, these two pathways give rise to a set of eight isomers (Scheme 1b).

**Fig. 2:**
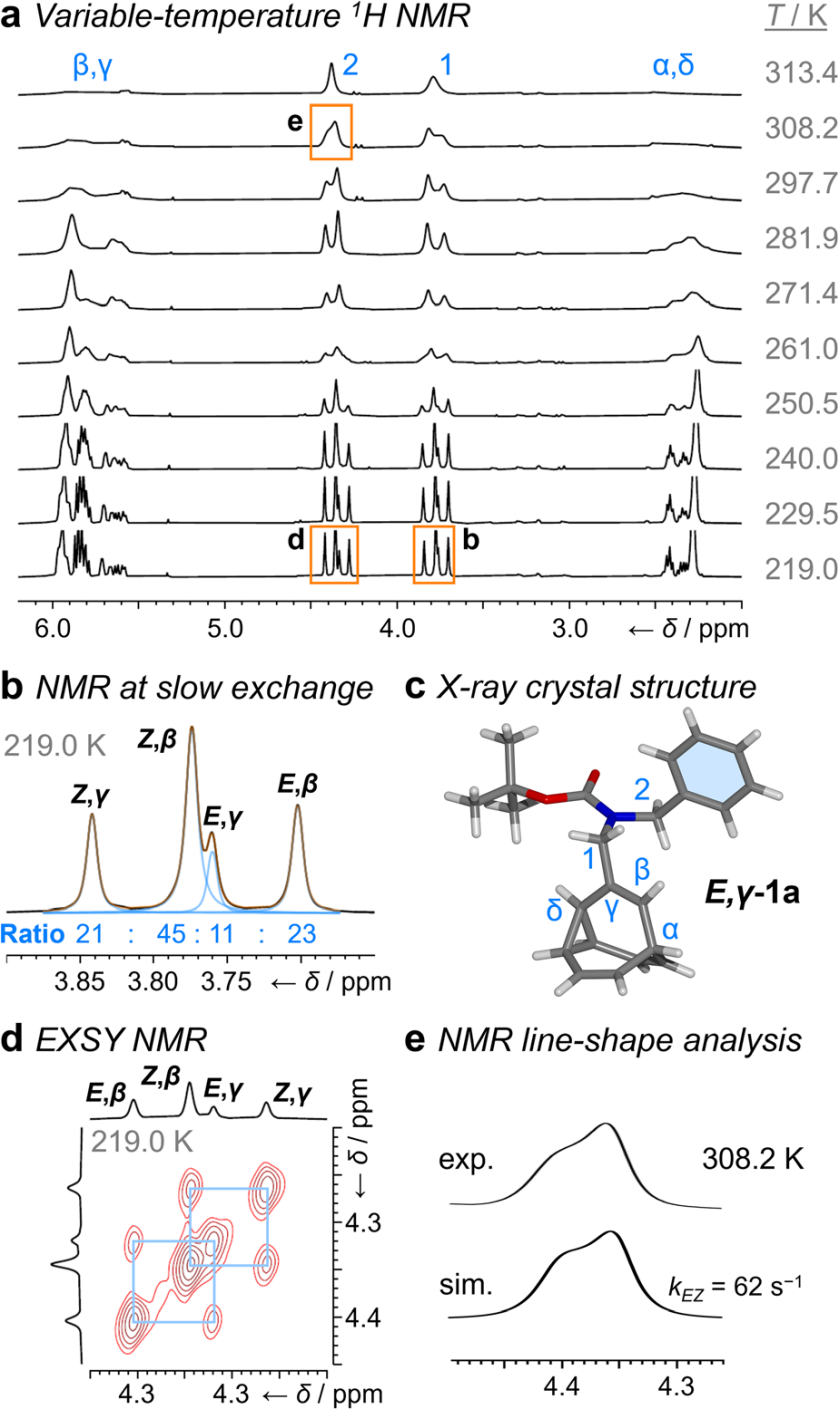
^1^H DNMR (499 MHz, CDCl_3_) and crystallographic analyses of 1a. (a) Partial ^1^H NMR spectra at temperatures ranging from 219 K to 313 K. Signals are assigned using the labels in Scheme 1. Regions in orange boxes correspond to those shown in panels b, d, and e. (b) Line fitting was used to deconvolute the H1 signals at 219 K. An overlay of the experimentally measured (black) and simulated (orange) traces is shown, including the integrals of the four simulated peaks (blue). (c) X-ray crystal structure of *E*,*γ*-1a. (d) Partial EXSY NMR spectrum (499 MHz, CDCl_3_, *t*_m_ = 500 ms). Blue boxes indicate cross peaks arising from Cope rearrangements. (e) Line-shape analysis for the H2 signal at 308 K, comparing the experimentally measured (exp.) and simulated (sim.) line shapes. See Table S1† for simulation parameters.

^1^H NMR Spectroscopic analysis of the benzyl derivative 1a in CDCl_3_ at 313 K (Fig. 2a) shows two broad resonances corresponding to the two sets of methylene protons, H1 and H2. Rapid chemical exchange at this temperature averages out the contributions from the different isomers to these two methylene resonances and causes the signals arising from the bullvalene methine groups, Hα–Hδ, to broaden and partially merge. At 219 K, the reduced rates of Cope rearrangements and carbamate rotation allow signals from the different species to be resolved.

Although there are eight possible isomers, the *α*- and *δ*-forms of mono-substituted bullvalenes are usually several kJ mol^−1^ higher in energy than the olefin-substituted *β*- and *γ*-forms,^16^ so they are not present in sufficient concentrations to be observed. *β*-Substitution is usually preferred over *γ*-substitution by ∼1–2 kJ mol^−1^.^2*c*^ In keeping with this expectation, line fitting of the H1 peaks indicates (Fig. 1b) that four isomers are present in a 45 : 23 : 21 : 11 ratio, which can be assigned to *Z*,*β*-1a, *E*,*β*-1a, *Z*,*γ*-1a, and *E*,*γ*-1a, respectively, using 2D NMR (Fig. S15–S22†). Shape-selective crystallization‡‡The energetics of crystal packing can outweigh the small solution-phase energetic bias towards certain isomers within fluxional mixtures.^2*e*,17^^2*e*,17^ from this mixture yields *E*,*γ*-1a (the least populated of the four isomers in solution) as the sole isomer in the solid state (Fig. 2c). In energetic terms (Table 1), the solution-phase population corresponds to small Gibbs energy biases Δ*G*_exp_ of ∼1.3 kJ mol^−1^ toward both *β*-isomers over their *γ*-substituted counterparts, and Δ*G*_exp_ of ∼1 kJ mol^−1^ for each *Z*-carbamate over its corresponding *E*-carbamate. Therefore, for 1a, there is no evidence that the *E*/Z-state of the molecule substantially changes the energetics of *β*/*γ*-isomerization, or *vice versa*. The two isomerization processes do not appear to be correlated.

**Table 1:** Populations and relative Gibbs energies of the isomers present in the dynamic mixtures and their transition states

State	1a, *p*/%			1a, Δ*G*/kJ mol^−1^			1b, *p*/%			1b, Δ*G*/kJ mol^−1^		
	*p*_exp_ a	*p*_calc1_ c	*p*_calcG_ d	Δ*G*_exp_^a^	Δ*G*_calc1_^f^	Δ*G*_calcG_^h^	*p*_exp_ a	*p*_calc1_ c	*p*_calcG_ d	Δ*G*_exp_^a^	Δ*G*_calc1_^f^	Δ*G*_calcG_^h^
*E*,*α*	—b	<0.1	<0.1	—b	25.5	25.4	—b	<0.1	<0.1	—b	34.5	34.0
*Z*,*α*	—b	<0.1	<0.1	—b	23.1	23.2	—b	<0.1	<0.1	—b	26.5	26.8
*E*,*β*	23	18	22	1.2	1.7	1.4	16	24	23	1.9	1.4	1.2
*Z*,*β*	45	47	46	0.0	0.0	1.0	27	18	28	1.0	1.5	0.8
*E*,*γ*	11	10	13	2.5	2.8	2.3	8	6	5	3.2	3.8	3.8
*Z*,*γ*	21	14	18	1.4	2.2	1.7	48	51	44	0.0	0.0	0.0
*E*,*δ*	—b	<1	<1	—b	8.7	8.2	—b	<0.1	<0.1	—b	14.8	14.1
*Z*,*δ*	—b	<1	1	—b	7.2	6.8	—b	<1	<1	—b	9.5	8.8
TS1	—	—	—	55.6	—g	—g	—	—	—	54.5	—g	—g
TS2	—	—	—	57.2	—g	—g	—	—	—	57.4	—g	—g
TS3	—	—	—	64.9^e^	—g	—g	—	—	—	54.8	—g	—g
TS4	—	—	—	—	—g	—g	—	—	—	55.3	—g	—g

a Measured at 219 K in CDCl_3_.

b The population of *α*- and *δ*-isomers is below the detection limit of the equipment used to acquire ^1^H NMR spectra.

c Population calculated as a Boltzmann distribution based on the DFT calculated energies of the most stable conformer for each constitutional and configurational isomer (Δ*G*_calc1_).

d Population calculated as a global Boltzmann distribution by summing the populations of all accessible conformers calculated for each constitutional and configurational isomer (Δ*G*_calcG_).

e Apparent Gibbs energy barrier for carbamate rotation (weighted average of TS3 and TS4) at 308 K.

f Relative Gibbs energies of only the most stable conformer for each isomer calculated at 219 K using PBE0-D3/def2-SV(P).^20*i*–*k*,*p*^

g Transition states were not modelled.

h Effective relative Gibbs energies based on the DFT calculated global Boltzmann distribution (*p*_calcG_) for the purpose of comparison with Δ*G*_exp_.

This system does, however, provide an unusual case of reversible covalent changes occurring in a molecule more rapidly than its bond rotation, *i.e.*, the carbamate isomerization of 1a proceeds at a slower rate than its Cope rearrangement. Using ^1^H–^1^H Exchange NMR Spectroscopy^18^ (EXSY) at 219 K (Fig. 2d), we found that the Cope rearrangements pass through transition states (TS1 and TS2, Scheme 1b) that are ∼55–57 kJ mol^−1^ above the lowest energy isomer, *Z*,*β*-1a, which is typical^19^ for bullvalene constitutional isomerization. There are no cross peaks visible between *E*- and *Z*-isomers at this temperature because of the slow carbamate rotation rate. At the higher temperature of 308 K, ^1^H NMR line-shape analysis (Fig. 2e) gives an averaged rate of carbamate rotation, *k*_*EZ*_, of 62 s^−1^, indicating that TS3 and TS4 lie almost 10 kJ mol^−1^ higher than TS1 at Δ*G*_exp_ of ∼65 kJ mol^−1^ relative to *Z*,*β*-1a (Table 1).

**Fig. 3:**
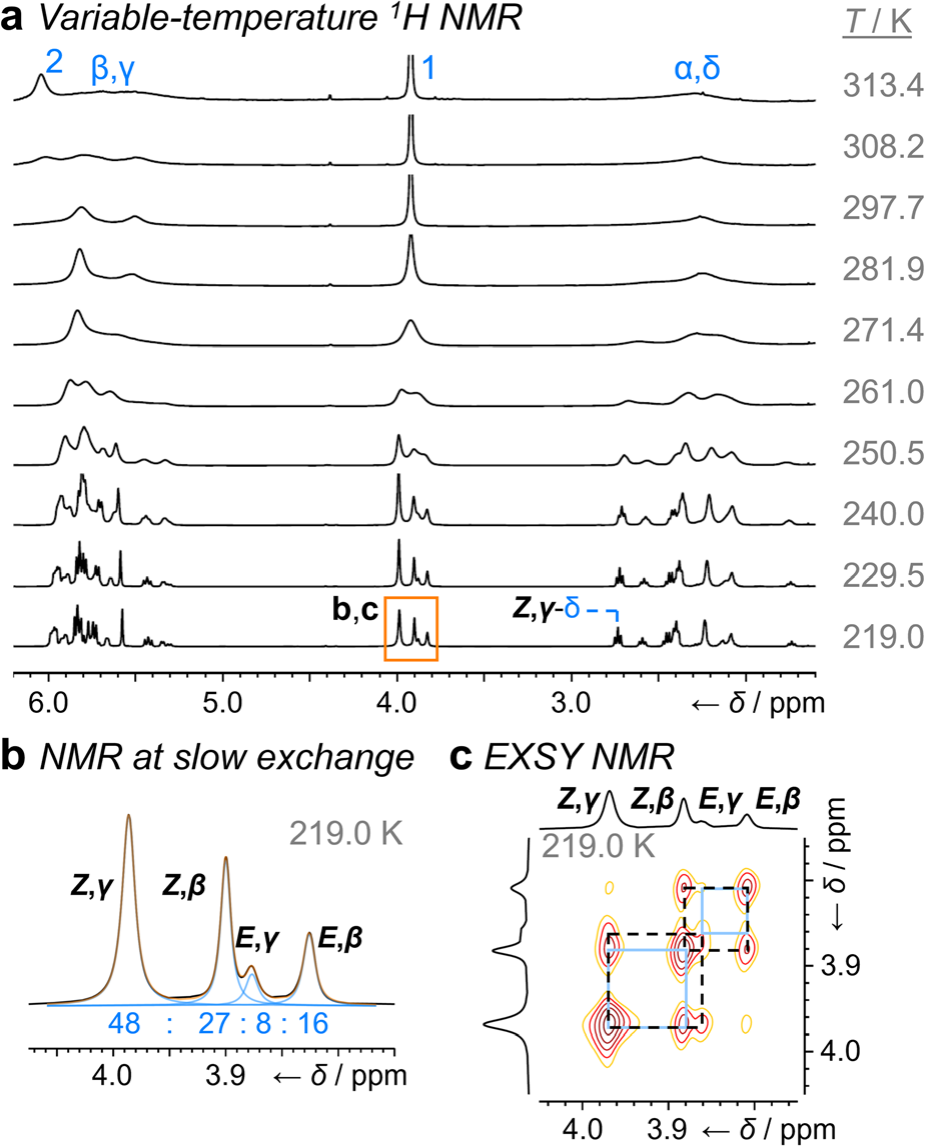
^1^H DNMR analysis (499 MHz, CDCl_3_) of 1b. (a) Partial ^1^H NMR spectra at temperatures ranging from 219 K to 313 K. Signals are assigned using the labels in Scheme 1. The region in an orange box corresponds to that shown in panels b and c. (b) Line fitting was used to deconvolute the H1 signals at 219 K. An overlay of the experimentally measured (black) and simulated (orange) traces is shown, including the integrals of the four simulated peaks (blue). (c) Partial EXSY NMR spectrum (499 MHz, CDCl_3_, *t*_m_ = 500 ms). Blue boxes indicate cross peaks arising from Cope rearrangements. Dashed black boxes indicate cross peaks arising from carbamate rotation.

**Fig. 4:**
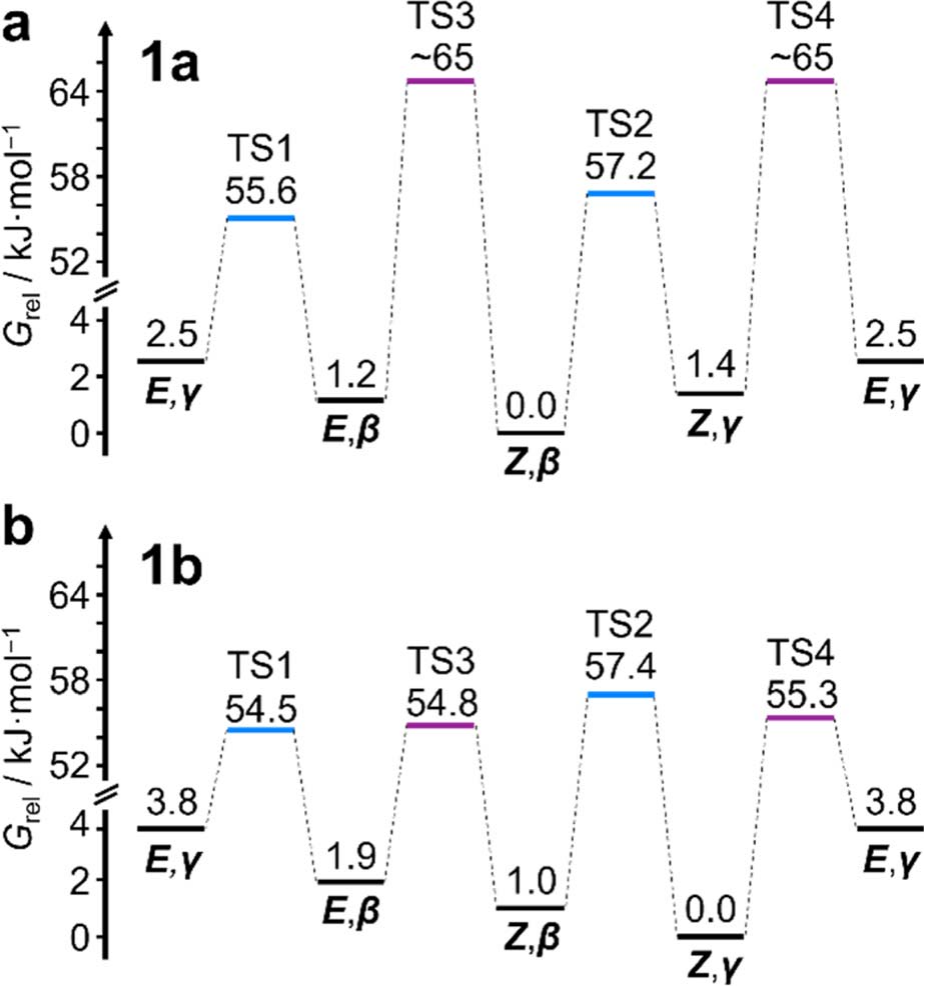
Potential energy diagrams for the observed isomers of (a) 1a and (b) 1b, showing the relative stabilization of *Z*,*γ*-1b. Transition states in blue correspond to Cope rearrangement steps, those in purple correspond to *E*/*Z*-isomerisation.

**Fig. 5:**
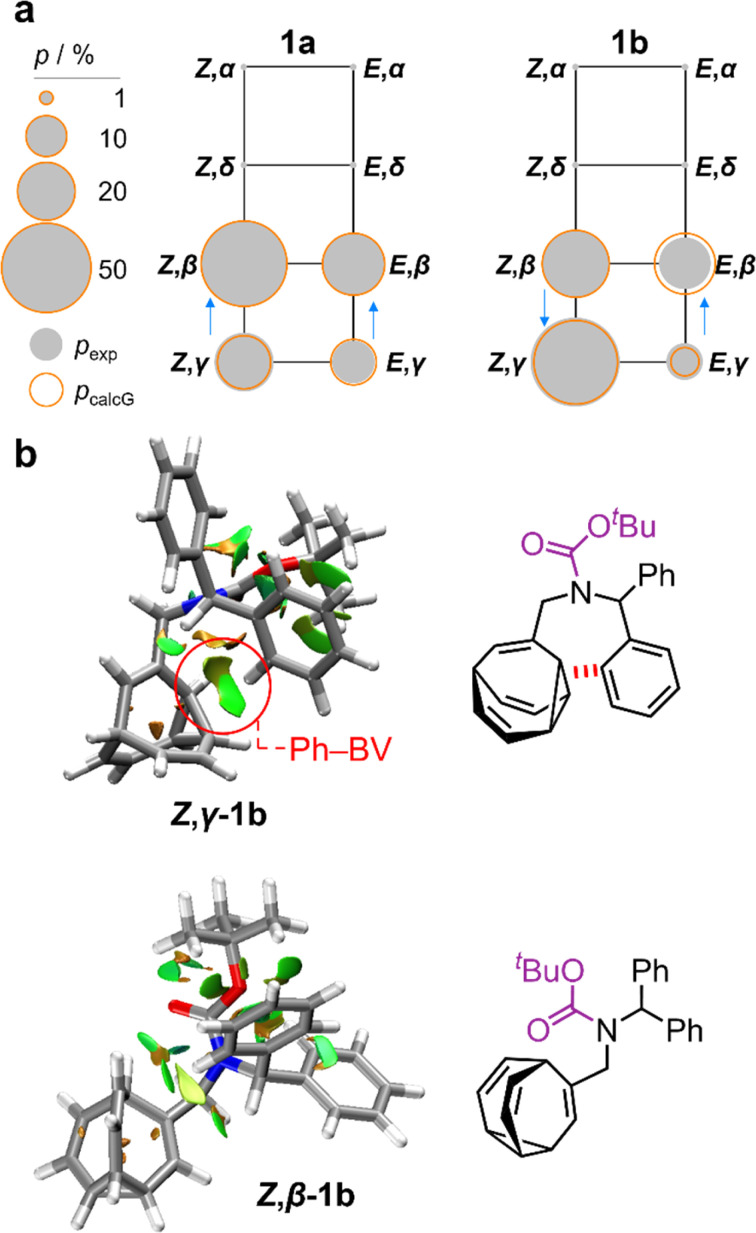
(a) Network diagrams for the isomerization of 1a and 1b showing the experimentally measured populations (*p*_exp_) of the *β*- and *γ*-isomers at 219 K as filled grey circles and the calculated populations (*p*_calG_) as hollow orange circles. According to DFT calculations, the *α*- and *δ*-isomers are present in <1%. Blue arrows point to the major species of each Cope rearrangement process, highlighting that the preference for the *β*- and *γ*-bullvalene isomers is correlated to the *E*-to-*Z* isomerization of 1b. (b) NCI plots [LC-PBE/def2-TZVP]^22^ of *Z*,*γ*-1b and *Z*,*β*-1b showing the increased Ph–BV interaction in *Z*,*γ*-1b.

Unlike 1a, the increased steric crowding present in the benzhydryl derivative 1b leads to correlated isomerization. The additional phenyl ring of 1b experiences long-range interactions with the bullvalene that influence the solution-phase isomerization equilibria. There are some key clues to this behavior in the variable-temperature NMR spectra (Fig. 3a). Crucially, the distribution of H1 methylene resonances (Fig. 3b) has been altered substantially relative to 1a. The *Z*,*β*-1b, *E*,*β*-1b, *Z*,*γ*-1b, and *E*,*γ*-1b isomers are present in a 27 : 16 : 48 : 8 ratio, as assigned by 2D NMR (Fig. S15–S22†). Therefore, the *Z*,*γ*-isomer is the most favored form of 1b, despite being only the third most populated isomer of 1a. The changes in relative Gibbs energy (*G*_rel_) between the 1a and 1b isomers is summarized graphically as a potential energy diagram in Fig. 4. A structural change from a hydrogen substituent to a phenyl group at a remote site has overridden the inherent preference^2*c*,16^ for the bullvalene *β*-isomer in the reaction network, but only when the carbamate is in its *Z*-form (Fig. 5a). The benzhydryl carbamate of 1b also rotates at a faster rate than the benzyl carbamate of 1a, exhibiting near-identical energy barriers of ∼54–58 kJ mol^−1^ for the four isomerization processes (Table 1).

To understand the unusual constitutional isomer distribution of 1b, we carried out DFT modelling. We generated all the constitutional isomers of 1a and 1b and carried out distance-geometry-based generation of their configurational and conformational isomers before optimizing the geometries at the PBE0-D3/def2-SV(P) level of theory.^1*e*,20^ As there are several rotatable single bonds in the structure of 1, each of the isomers observed by low-temperature NMR itself represents a rapidly interconverting population of conformers. The relative energies of all the isomers are tabulated in Tables S3 and S4.† Cartesian coordinates are available in the ESI.† To take one representative example, there are six conformers of *Z*,*β*-1a spanning a range of ∼16 kJ mol^−1^ in energy, of which the two lowest energy structures are within just 2.5 kJ mol^−1^ of one another.

Despite there being numerous different structures contributing to the overall energetics of *E*-to-*Z* and *β*-to-*γ* isomerization, the experimentally observed populations, *p*_exp_, can be approximated reasonably well by analyzing just the lowest energy conformers (Table 1). For 1a, the calculated energies, Δ*G*_calc1_, of the lowest energy conformer for each of the four observable isomers gives a Boltzmann distribution, *p*_calc1_, that matches closely to *p*_exp_. For 1b, however, this simplified approach incorrectly predicts that more of *E*,*β*-1b is present than *Z*,*β*-1b. Instead, it is necessary to perform a global Boltzmann population analysis (giving *p*_calcG_) that takes into account all the accessible conformers in order to reproduce the experimentally observed hierarchy of isomers. This observation indicates that for bullvalene derivatives with several rotatable single bonds, accurate modelling of the shapeshifting rearrangements requires consideration of the full energetic landscape of accessible conformers.

Noncovalent interaction (NCI) plots^21^ generated using the DFT-optimized geometries reveal (Fig. 5b and S26–S29†) the interactions that are responsible for the correlated isomerization of 1b. In addition to several close contacts between the crowded *tert*-butyl, carbonyl, and phenyl groups, which are present in all of the isomers, the lowest energy conformer of *Z*,*γ*-1b shows evidence of substantial interaction between an *ortho*-hydrogen of one phenyl ring with the bullvalene cyclopropyl ring (Ph–BV interaction, Fig. 5b). This long-range interaction is less significant in the NCI analyses of the other 1b isomers (Fig. S26–S29†), such as *Z*,*β*-1b (Fig. 5b). Experimental evidence for the interaction in *Z*,*γ*-1b is apparent in its low-temperature ^1^H NMR spectrum (Fig. 3a) as its Hδ resonance is shifted downfield relative to the Hδ signals of the other isomers of 1b, which is indicative of deshielding caused by the aromatic ring current. The NOESY NMR spectrum (Fig. S23†) also confirms Hδ undergoes through-space interaction with a phenyl group. Although this Ph–BV interaction appears to be a relatively weak van der Waals contact, the combination of it together with other subtle differences in the attractive and repulsive noncovalent bonding interactions present in 1b is evidently sufficient to provide enough of an energetic bias to overcome the ∼1–2 kJ mol^−1^ preference for the *β*-isomer.

## Conclusions

In summary, the shapeshifting constitutional rearrangements of even structurally simple bullvalenes can be influenced by a complementary, remote isomerization process. Given that the energetic differences between bullvalene isomers are often as little as a few kJ mol^−1^, relatively subtle noncovalent bonding interactions, including weak van der Waals contacts, are sufficient to reshuffle the proportions of each isomer. In the system presented here, the introduction of an additional phenyl ring to the structure of 1a causes the *Z*,*γ*-isomer to change from being the third most populous to being the major species of 1b as a result of a Ph–BV interaction. Programming in switchable long-range interactions may allow for control to be exercised over the large number of constitutional isomers that arise in multi-substituted bullvalene derivatives.

## Author contributions

Conceptualization: BAH, PRM. Investigation: BAH, WM, MKR, ANB, JAA, CR. Writing: BAH, WM, ANB, YW, RA, PRM.

## Conflicts of interest

There are no conflicts to declare.

## Supplementary Material

SC-015-D4SC03699A-s001

SC-015-D4SC03699A-s002

SC-015-D4SC03699A-s003

## Data Availability

Experimental synthetic procedures, characterization data and theoretical calculation results are available in the ESI.†
