# Supplementary material for: Correlated shapeshifting and configurational isomerization
Source: Chem Sci. 2024 Aug 23;15(36):14618–24. doi: 10.1039/d4sc03699a (PMC11370815; doi:10.1039/d4sc03699a)
Supplement: SC-015-D4SC03699A-s001 [file SC-015-D4SC03699A-s001.pdf]

Supplementary Information (SI) for Chemical Science.
This journal is © The Royal Society of Chemistry 2024

Correlated Shapeshifting and Configurational Isomerization

Burhan A. Hussein,[a] William Maturi,[a,b] Mary Kate Rylands,[a] Aisha N. Bismillah,[a,b] Yuzhen Wen,[a,b] Juan A. Aguilar,[a] Rabia Ayub,[b] Conor D. Rankine,[b] and Paul R. McGonigal*[a,b]

[a] *Department of Chemistry, Durham University, Lower Mountjoy, Stockton Road, Durham, DH1 3LE (UK).*

[b] *Department of Chemistry, University of York, Heslington, York, YO10 5DD (UK).*

E-mail: paul.mcgonigal@york.ac.uk

Table of Contents

1. General Methods S1

2. Synthetic Procedures S3

3. ^{1}H and ^{13}C NMR Spectroscopic Characterization S12

4. Structural Assignment by 1D and 2D NMR S26

4.1. Benzhydryl Boc Bullvalene (**1b**) S26

4.2. Benzyl Boc Bullvalene (**1a**) S30

5. NOESY NMR Analysis S34

6. X-Ray Crystallographic Analysis S35

7. Computational Details S37

7.1. Conformer Generation S37

7.2. DFT Geometry Optimization S38

7.3. NCI Plots S43

8. References S47

Supporting Information

1. General Methods

Materials:

All reagents were purchased from commercial suppliers (Sigma-Aldrich, Acros Organics, or Alfa Aesar) and used without further purification. Pre-packed SiO_2 and high-performance liquid chromatography (HPLC) SiO_2 columns were purchased from Teledyne Isco and were used without further modifications. Analytical thin-layer chromatography (TLC) was performed on neutral aluminium sheet SiO_2 gel plates and visualised under ultraviolet (UV) irradiation (254 nm).

Instrumentation and Analytical Techniques:

Nuclear magnetic resonance (NMR) spectra were recorded using a Bruker Advance (III)-400 (^{1}H 400.130 MHz and ^{13}C 100.613 MHz), Jeol ECS-400(A) (^{1}H 399.760 MHz and ^{13}C 150.903 MHz), Jeol ECS-400(D) (^{1}H 399.830 MHz and ^{13}C 100.530 MHz), Bruker AVIIIHD-500 (^{1}H 500.230 MHz and ^{13}C 125.782 MHz), Varian Inova-500 (^{1}H 500.130 MHz and ^{13}C 125.758 MHz), or a Varian VNMRS-700 (^{1}H 700.130 MHz and ^{13}C 176.048 MHz) spectrometer at a constant temperature of 298 K unless otherwise stated. For variable-temperature (VT) measurements, operating temperatures were calibrated using an internal calibration solution of MeOH and glycerol. Chemical shifts (δ) are reported in parts per million (ppm) relative to the signals corresponding to residual non-deuterated solvents [$CDCl_3$: $\delta = 7.26$ or 77.16]. Coupling constants (J) are reported in Hertz (Hz). ^{13}C NMR experiments were proton decoupled. Assignments of ^{1}H and ^{13}C NMR signals were accomplished by one-dimensional (1D) and two-dimensional (2D) NMR spectroscopy (COSY, NOESY, HSQC, HMBC, EXSY, ROESY and HSQC-TOCSY). NMR spectra were processed using MestReNova version 14.0. Data are

reported as follows: chemical shift; multiplicity; coupling constants; integral and assignment. Low-resolution Atmospheric Solids Analysis Probe (ASAP)-mass spectrometry (MS) were performed using a Waters Xevo QTOF equipped with an ASAP. High-resolution (HR) electrospray ionisation (HR-ESI) and ASAP (HR-ASAP) mass spectra were measured using a Waters LCT Premier XE high resolution, accurate mass UPLC ES MS (also with ASAP ion source). Melting points were recorded using a Gallenkamp (Sanyo) apparatus and are uncorrected. Photoirradiation reactions were performed with a 365 nm UV light (3.4 array of surface mounted 365 nm light-emitting diodes (LEDs)). The X-ray single crystal data for compound ***E*,*γ*-1a** were collected at 120.0(2) K using CuKα radiation (λ = 1.54178Å) on a Bruker D8Venture (Photon III MM C7 CPAD detector, IµS-microsource, focusing mirrors, 1° ω-scan, shutterless mode, 8-25 s variable exposure time) 3-circle diffractometer equipped with a Cryostream-700 (Oxford Cryosystems) open-flow N_2 cryostat. The structure was solved by direct method and refined by full-matrix least squares on F^2 for all data using Olex2[S1] and SHELXTL[S2] software. All non-hydrogen atoms were refined in anisotropic approximation, hydrogen atoms were refined isotropically. Crystal data and parameters of refinement are listed in Table S2. Crystallographic data for the structure have been deposited with the Cambridge Crystallographic Data Centre as supplementary publication CCDC-2294194.

2. Synthetic Procedures

***tert*-Butyl benzyl(prop-2-yn-1-yl)carbamate (4a)**

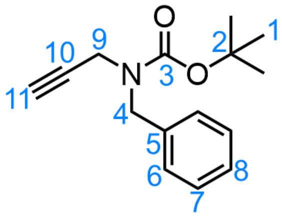

To a single-necked round-bottomed flask was added CH_2Cl_2 (20 mL). The solvent was cooled to 0 °C before the addition of Et_3N (2.48 g, 24.5 mmol), benzylamine (2.19 g, 20.4 mmol) and boc anyhdride (4.90 g, 22.4 mmol) in a sequential manner. The mixture was stirred at rt for 24 h. The reaction was quenched with H_2O (50 mL) and the aqueous layer was extracted with CH_2Cl_2 (3 × 20 mL). The combined organic phases were dried over anhydrous $MgSO_4$, and the solvent was removed under reduced pressure to give ^{t}butyl benzylcarbamate as a colourless oil. The crude ^{t}butyl benzylcarbamate (3.79 g, 18.3 mmol) was added to a two-necked round-bottomed flask under an Ar atmosphere and dissolved in anhydrous DMF (30 mL). The mixture was cooled to 0 °C and NaH (658 mg, 27.4 mmol) was added. After stirring at this temperature for 30 min, propargyl bromide (3.2 mL, 36.6 mmol) was added, the mixture was warmed to 20 °C followed by stirring for 5 h. The reaction was quenched with a saturated aqueous solution of KOH (2 M, 30 mL), then extracted with EtOAc (3 × 30 mL). The combined organic layers were dried over anhydrous $MgSO_4$, filtered and the solvent was removed under reduced pressure. The crude residue purified by column chromatography (Teledyne Isco CombiFlash Rf+ system, 24 g, SiO_2, hexanes−EtOAc, 0–100% gradient elution) affording the title compound as a yellow oil (3.15 g, 12.8 mmol, 70%).**^{1}H NMR** (400 MHz, $CDCl_3$, 298 K) δ 7.35–7.27 (m, 5H, H_6, H_7 and H_8), 4.55 (s, 2H, H_4), 3.90 (s, 2H, H_9), 2.21 (s, 1H, H_{11}), 1.49 (s, 9H, H_1). **HRMS-ASAP** *m*/*z* = 246.1213 $[M+H]^+$, calculated for $C_{15}H_{20}NO_2^+$ = 246.1494. Spectroscopic data were consistent with those published previously.[S3]

***tert*-Butyl benzhydryl(prop-2-yn-1-yl)carbamate (4b)**

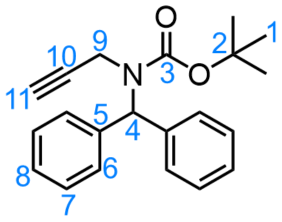

To a single-necked round-bottomed flask was added CH_2Cl_2 (20 mL). The solvent was cooled to 0 °C before the addition of Et_3N (2.48 g, 24.5 mmol), benzyhydrylamine (3.74 g, 20.4 mmol) and boc anyhdride (4.90 g, 22.4 mmol) in a sequential manner. The reaction was stirred at rt for 24 h. The reaction was quenched with H_2O (50 mL) and the aqueous layer was extracted with CH_2Cl_2 (3 × 20 mL). The combined organic phases were dried over anhydrous $MgSO_4$, and the solvent was removed under reduced pressure to give ^{t}butyl benzhydrylcarbamate as a colourless oil. A portion of the crude ^{t}butyl benzhydrylcarbamate (5.18 g, 18.3 mmol) was added to a two-necked round-bottomed flask under an Ar atmosphere and dissolved in anhydrous DMF (30 mL). The mixture was cooled to 0 °C and NaH (658 mg, 27.4 mmol) was added. After stirring at this temperature for 30 min, propargyl bromide (3.2 mL, 36.6 mmol) was added, the mixture was warmed to 20 °C followed by stirring for 5 h. The reaction was quenched with a saturated aqueous solution of KOH (2 M, 30 mL), then extracted with EtOAc (3 × 30 mL). The combined organic layers were dried over anhydrous $MgSO_4$, filtered and the solvent was removed under reduced pressure. The crude residue purified by column chromatography (Teledyne Isco CombiFlash Rf+ system, 24 g, SiO_2, hexanes−EtOAc, 0–100% gradient elution) affording the title compound as a yellow oil (4.15 g, 13.4 mmol, 64%). **^{1}H NMR** (400 MHz, $CDCl_3$, 298 K) δ 7.42–7.23 (m, 10H, H_6, H_7 and H_8), 6.48 (s, 1H, H_4), 3.92 (s, 2H, H_9), 2.04 (s, 1H, H_{11}), 1.43 (s, 9H, H_1). **^{13}C NMR** (176 MHz, $CDCl_3$, 298 K) δ 155.3 (C_3), 139.8 (C_5), 128.8 (C_7), 128.4 (C_8), 127.5 (C_6), 80.9 (C_{11}), 80.6 (C_2), 70.5 (C_{10}), 63.9 (C_4), 35.0 (C_9), 28.6 (C_1). **HRMS-ESI** *m*/*z* = 344.1615 $[M+Na]^+$, calculated for $C_{21}H_{23}NNaO_2^+$ = 344.1621.

tert-Butyl-_N_-benzyl-_N_-(((2_Z_,4_Z_)-bicyclo[4.2.2]deca-2,4,7,9-tetraen-7-yl)methyl)carbamate (5a)

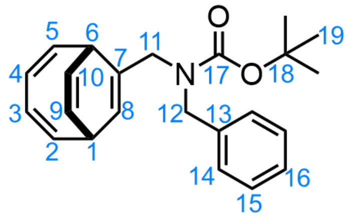

ZnI_2 (61.3 mg, 0.192 mmol) was added to an oven-dried microwave vial and heated to 280 °C under vacuum for 5 min. $CoBr_2$(dppe) (dppe = 1,2-bis(diphenylphosphino)ethane) (59.3 mg, 0.288 mmol) and activated Zn dust (18.8 mg, 0.096 mmol) were added to the same vial. The microwave vial was sealed and the mixture deoxygenated (3 × N_2 purge, followed by an evacuation cycle). Anhydrous 2,2,2-trifluoroethanol (1.5 mL) was added and this was also followed by deoxygenation of the mixture (3 × freeze-pump-thaw cycles under N_2). The mixture was stirred for 15 min at rt, followed by the addition of cyclooctatetraene (0.11 mL, 0.960 mmol). Next, *tert*-butyl benzyl(prop-2-yn-1-yl)carbamate (**4a**) (0.38 mL, 1.44 mmol) was added dropwise over 1.5 h, after which the reaction mixture was stirred for 22 h at 55 °C. The reaction mixture was filtered through a SiO_2 plug and eluted with EtOAc with the solvent being removed under reduced pressure. The crude residue purified by column chromatography (Teledyne Isco CombiFlash Rf+ system, 12 g, SiO_2, hexanes–EtOAc, 0–30% gradient elution) affording the title compound as a colourless oil (185 mg, 0.53 mmol, 55%). NMR spectroscopy shows a mixture (approximately 1:1) of carbamate *E*/*Z* isomers. Where possible, the ^{1}H and ^{13}C NMR resonances of the title compound have been assigned as either the *E* or *Z* isomer based on 2D spectra. Some *E*/*Z* isomer assignments are not possible due to signal overlap. **^{1}H NMR** (700 MHz, $CDCl_3$, 298 K) δ 7.30 – 7.22 (m, 2H, H_{15}), 7.22 – 7.16 (m, 2H, H_{14}), 7.13 (br. s, 1H, H_{16}), 6.28 – 6.17 (m, 1H, H_2), 6.14 – 6.04 (m, 1H, H_5), 5.79 – 5.75 (m, 1H, H_3 or H_4), 5.75 – 5.72 (m, 1H, H_3 or H_4), 5.67 – 5.61 (m, 1H, H_{10}), 5.60 – 5.56 (m, 1H, H_9), 5.48 – 5.38

(m, 1H, H_8), 4.34 – 4.06 (m, 2H, H_{12}), 3.88 – 3.64 (m, 1H, H_{11}), 3.41 – 3.31 (m, 0.5H, H_{6E} or H_{6Z}), 3.27 – 3.20 (m, 0.5H. H_{6E} or H_{6Z}), 3.19 – 3.06 (m, 1H, H_1), 1.55 – 1.37 (m, 9H, H_{19}). **^{13}C NMR** (176 MHz, $CDCl_3$, 298 K) δ 155.8 (C_{17}), 141.7 (C_{5E} or C_{5Z}), 141.6 (C_{2E} or C_{2Z}), 141.4 (C_{2E} or C_{2Z}), 141.0 (C_{5E} or C_{5Z}), 138.4 (C_{13E} or C_{13Z}), 138.2 (C_{13E} or C_{13Z}), 132.2 (C_{7E} or C_{7Z}), 131.6 (C_{7E} or C_{7Z}), 128.4 (C_{15}), 128.1 (C_{14E} or C_{14Z}), 127.6 (C_{16}), 127.1 (C_{14E} or C_{14Z}), 124.8 (C_{3E} or C_{3Z} or C_{4E} or C_{4Z}), 124.5 (C_{3E} or C_{3Z} or C_{4E} or C_{4Z}), 124.4 (2C, C_{3E} or C_{3Z} or C_{4E} or C_{4Z}), 121.1 (2C, C_{9E} or C_{9Z} or C_{10E} or C_{10Z}), 121.0 (C_{9E} or C_{9Z} or C_{10E} or C_{10Z}), 120.9 (C_{9E} or C_{9Z} or C_{10E} or C_{10Z}), 120.7 (C_{8E} or C_{8Z}), 120.1 (C_{8E} or C_{8Z}), 79.8 (C_{18}), 48.5 (C_{12E} or C_{12Z}), 48.4 (2C, C_{11E} and C_{11Z}), 48.1 (C_{12E} or C_{12Z}), 36.9 (C_{6E} or C_{6Z}), 36.7 (C_{6E} or C_{6Z}), 35.0 (C_{1E} or C_{1Z}), 34.9 (C_{1E} or C_{1Z}), 28.5 (3C, C_{19}). **HRMS ASAP** *m*/*z* = 350.2120 $[M+H]^+$, calculated for $C_{23}H_{28}NO_2^+$: 350.2120.

tert-Butyl-*N*-benzhydrl-*N*-(((2*Z*,4*Z*)-bicyclo[4.2.2]deca-2,4,7,9-tetraen-7-yl)methyl) carbamate (5b)

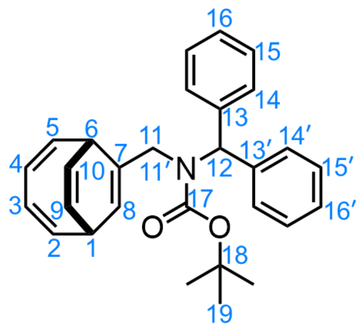

ZnI_2 (61.3 mg, 0.19 mmol) was added to an oven-dried microwave vial and heated to 280 °C under vacuum for 5 min. $CoBr_2$(dppe) (dppe = 1,2-bis(diphenylphosphino)ethane) (59.3 mg, 0.29 mmol) and activated Zn dust (18.8 mg, 0.10 mmol) were added to the same vial. The microwave vial was sealed and the mixture deoxygenated (3 × N_2 purge, followed by an evacuation cycle). Anhydrous 2,2,2-trifluoroethanol (1.5 mL) was added and this was also followed by deoxygenation of the mixture (3 × freeze-pump-thaw cycles under N_2). The mixture was stirred for 15 min at rt,

followed by the addition of cyclooctatetraene (0.11 mL, 0.960 mmol). Next, *tert*-butyl benzhydryl(prop-2-yn-1-yl)carbamate (**4b**) (462 mg, 1.44 mmol) was added dropwise over 1.5 h, after which the reaction mixture was stirred for 22 h at 70 °C. The reaction mixture was filtered through a SiO_2 plug and eluted with EtOAc with the solvent being removed under reduced pressure. The crude residue purified by column chromatography (Teledyne Isco CombiFlash Rf+ system, 12 g, SiO_2, hexanes–EtOAc, 0–30% gradient elution) affording the title compound as a colourless oil (194 mg, 0.26 mmol, 50%). NMR spectroscopy shows a mixture (approximately 1:1) of carbamate *E*/*Z* isomers. Where possible, the ^{1}H and ^{13}C NMR resonances of the title compound have been assigned as either the *E* or *Z* isomer based on 2D spectra. Some *E*/*Z* isomer assignments are not possible due to signal overlap. Prime symbols are also used in the spectral assignment of the title compound to label diastereotopic positions. **^{1}H NMR** (400 MHz, $CDCl_3$, 298 K) δ 7.37 – 7.22 (m, 6H, H_{15}, $H_{15'}$, H_{16} and $H_{16'}$), 7.18 (d, *J* = 7.4 Hz, 2H, H_{14} or $H_{14'}$), 7.13 (d, *J* = 7.3 Hz, 2H, H_{14} or $H_{14'}$), 6.17 (br. s, 1H, H_8), 6.14 (td, *J* = 8.8, 3.1 Hz, 1H, H_5), 6.05 – 5.92 (m, 1H, H_2), 5.71 (d, *J* = 8.2 Hz, 1H, H_3 or H_4), 5.69 – 5.63 (m, 1H, H_3 or H_4), 5.62 – 5.56 (m, 1H, H_9 or H_{10}), 5.54 (dd, *J* = 8.6, 5.6 Hz, 1H, H_9 or H_{10}), 5.11 (s, 0.5H, H_{12E} or H_{12Z}), 5.10 (s, 0.5H, H_{12E} or H_{12Z}), 4.42 – 4.12 (m, 1H, H_{11} and $H_{11'}$), 3.63 (d, 1H, H_{11} and $H_{11'}$), 3.14 (s, 1H, H_1), 3.00 (q, *J* = 6.8 Hz, 1H, H_6), 1.33 (s, 9H, H_{19}). **^{13}C NMR** (101 MHz, $CDCl_3$, 298 K) δ 156.2 (C_{17}), 141.8 (C_2), 141.3 (C_5), 140.9 (C_8), 140.0 (2C, C_{13} and $C_{13'}$), 132.2 (C_{12E} or C_{12Z}), 132.0 (C_{12E} or C_{12Z}), 129.0 (2C, C_{14} and $C_{14'}$), 128.3 (C_{15} or $C_{15'}$), 128.2 (C_{15} or $C_{15'}$), 127.2 (C_{16} or $C_{16'}$), 127.0 (C_{16} or $C_{16'}$), 124.7 (C_3), 124.4 (C_4), 121.0 (C_9 or C_{10}), 120.9 (C_9 or C_{10}), 119.1 (C_7), 80.2 (C_{18}), 63.9 (C_{11} or $C_{11'}$), 36.3 (C_1), 34.9 (C_6), 28.4 (C_{19}). **HRMS ASAP** *m*/*z* = 426.2429 $[M+H]^+$, calculated for $C_{29}H_{32}NO_2^+$: 426.2432.

Benzyl Bullvalene (1a)

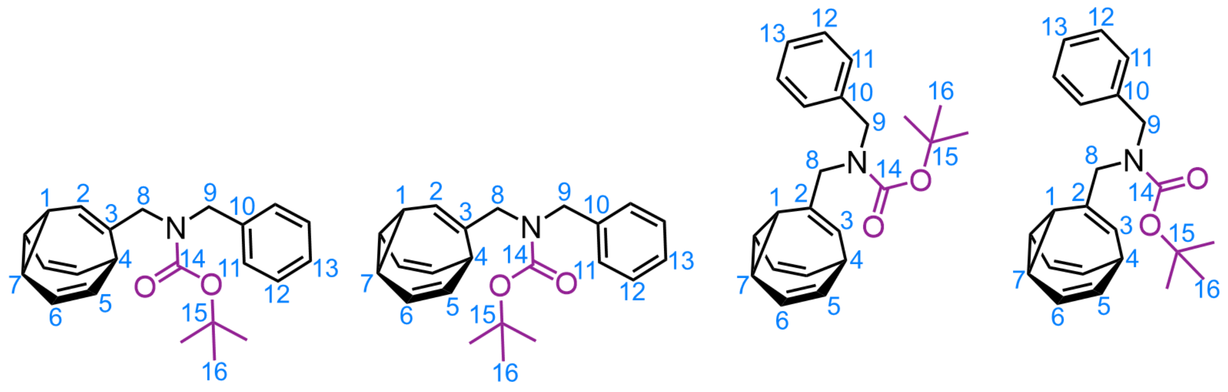

Isomer Ratio

***Z,β*-1a** : ***E,β*-1a** : ***Z,γ*-1a** : ***E,γ*-1a**

45 : 23 : 21 : 11

In an oven-dried microwave vial under an Ar atmosphere, *tert*-butyl-*N*-benzyl-*N*-(((2*Z*,4*Z*)-bicyclo[4.2.2]deca-2,4,7,9-tetraen-7-yl)methyl)carbamate (**5a**) (180 mg, 0.51 mmol) and thioxanthene-9-one (1 mg, 0.01 mmol) were dissolved in anhydrous THF (1.2 mL). The mixture was stirred for 3 h, while being irradiated with 365 nm UV light (3.4 array of surface mounted 365 nm LEDs) at rt. The solvent was removed under reduced pressure and the crude residue purified by column chromatography (Teledyne Isco CombiFlash Rf+ system, 8 g, SiO_2, hexanes–EtOAc, 0–10% gradient elution, followed by 12 g HPLC SiO_2, hexanes–CH_2Cl_2, 0–100% gradient elution) affording the title compound as a colourless solid (90 mg, 0.26 mmol, 50%). **M. P.** 130–131 °C. Where possible, the ^{1}H and ^{13}C NMR resonances of the title compound have been assigned as either the *E* or *Z* isomer as well as the *β* or *γ* isomer based on 2D spectra. Some assignments are not possible due to signal overlap. **^{1}H NMR** (500 MHz, $CDCl_3$, 219 K) δ 7.39 – 7.31 (m, 2H, H_{12}), 7.31 – 7.27 (m, 1H, H_{13}), 7.25 – 7.15 (m, 2H, H_{11}), 6.00 – 5.89 (m, 2H, H_6), 5.89 – 5.77 (m, 2H, H_5), 5.71 (s, 0.38H, *Z,β*-H_2), 5.66 (s, 0.22H, *E,β*-H_2), 5.62 (d, *J* = 8.8 Hz, 0.21H, *Z,γ*-H_3), 5.58 (d, *J* = 8.4 Hz, 0.22H, *E,γ*-H_3), 4.40 (s, 0.42H,

E,β-H_9), 4.35 (s, 0.72H, *Z,β*-H_9), 4.32 (s, 0.27H, *E,γ*-H_9), 4.26 (s, 0.37H, *Z,γ*-H_9), 3.82 (s, 0.38H, *Z,γ*-H_8), 3.78 – 3.73 (m, 0.96H, *Z,β*-H_8 and *E,γ*-H_8), 3.69 (s, 0.42H, *E,β*-H_8), 2.48 – 2.34 (m, 1H, H_4), 2.34 – 2.24 (m, 3H, H_1 and H_7), 1.48 (s, 3.06H, *E,β*-H_{16} and *E,γ*-H_{16}), 1.46 (s, 5.94H, *Z,β*-H_{16} and *Z,γ*-H_{16}). **^{13}C NMR** (126 MHz, $CDCl_3$, 219 K) δ 156.4 (*E,β*-C_{14} or *Z,γ*-C_{14}), 156.3 (*Z,β*-C_{14}), 156.2 (*E,γ*-C_{14}), 156.1 (*E,β*-C_{14} or *Z,γ*-C_{14}), 138.1 (*Z,γ*-C_{10}), 138.0 (*Z,β*-C_{10}), 137.9 (*E,β*-C_{10}), 137.8 (*E,γ*-C_{10}), 137.0 (*Z,β*-C_2), 136.6 (*E,β*-C_2), 134.5 (*Z,γ*-C_3), 134.0 (*E,γ*-C_3), 128.8 (C_{12}), 128.7 (C_{12}), 128.6 (C_{12}), (128.1–127.2 C_{13}, C_{11}, C_6 and C_5), 128.1, 127.9, 127.9, 127.8, 127.8, 127.7, 127.6, 127.5, 127.5, 127.4, 127.4, 127.3, 127.2, 125.2 (*Z,γ*-C_2), 124.9 (*Z,β*-C_3), 124.7 (*E,γ*-C_2), 123.6 (*E,β*-C_3), 80.4 (C_{14}), 80.4 (C_{14}), 80.4 (C_{14}), 53.7 (*E,γ*-C_8), 53.3 (*Z,γ*-C_8), 52.4 (*E,β*-C_8), 52.0 (*Z,β*-C_8), 47.4 (*Z,γ*-C_9), 47.1 (*Z,β*-C_9), 46.9 (*E,β*-C_9), 46.8 (*E,γ*-C_9), 31.8 (*Z,β*-C_1), 31.8 (*Z,γ*-C_1), 30.0 (*E,β*-C_1), 30.0 (*E,γ*-C_1), 28.6 (C_{16}), 28.6 (C_{16}), 28.5 (C_{16}), 21.1 (C_7), 20.9 (C_7), 20.7 (C_7), 20.1 (C_7), 20.1 (C_7), 19.9 (C_7), 19.8 (C_7). **HRMS ASAP** *m/z* = 350.2137 $[M+H]^+$, calculated for $C_{23}H_{28}NO_2^+$: 350.2120.

Benzhydrlyl Bullvalene (1b)

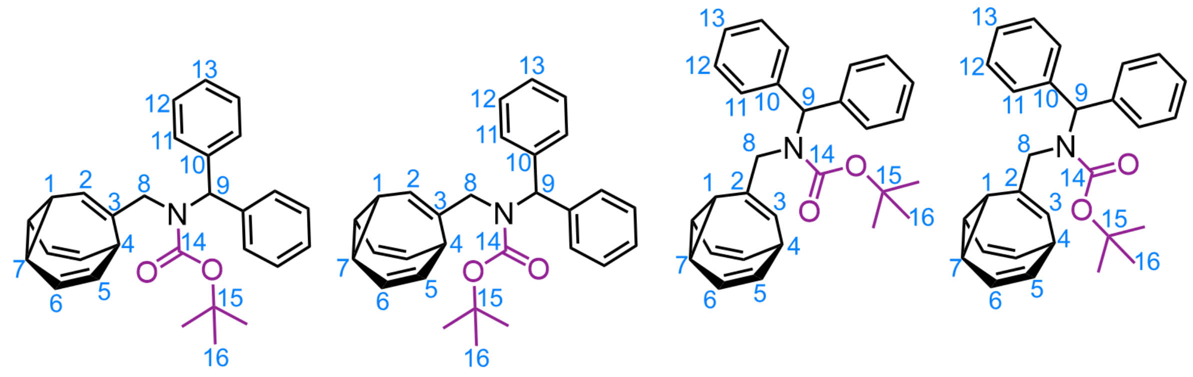

***Z,β*-1b** ***E,β*-1b** ***Z,γ*-1b** ***E,γ*-1b**

Isomer Ratio
***Z,β*-1b : *E,β*-1b : *Z,γ*-1b : *E,γ*-1b**
16 : 27 : 48 : 8

In an oven-dried microwave vial under an Ar atmosphere, *tert*-butyl-*N*-benzhydrl-*N*-(((2*Z*,4*Z*)-bicyclo[4.2.2]deca-2,4,7,9-tetraen-7-yl)methyl) carbamate (**5b**) (140 mg, 0.28 mmol) and thioxanthene-9-one (1 mg, 0.01 mmol) were dissolved in anhydrous THF (1.2 mL). The mixture was stirred for 3 h, while being irradiated with 365 nm UV light (3.4 array of surface mounted 365 nm LEDs) at rt. The solvent was removed under reduced pressure and the crude residue purified by column chromatography (Teledyne Isco CombiFlash Rf+ system, 8 g, SiO_2, hexanes–EtOAc, 0–10% gradient elution, followed by 12 g HPLC SiO_2, hexanes–CH_2Cl_2, 0–100% gradient elution) affording the title compound as a colourless wax (63 mg, 0.15 mmol, 54%). Where possible, the ^{1}H and ^{13}C NMR resonances of the title compound have been assigned as either the *E* or *Z* isomer as well as the *β* or *γ* isomer based on 2D spectra. Some assignments are not possible due to signal overlap. Prime symbols are also used in the spectral assignment of the title compound to label diastereotopic positions. **^{1}H NMR** (500 MHz, $CDCl_3$, 219 K) δ 7.37 – 7.26 (m, 6H, H_{12}, H_{13}), 7.22 – 7.14 (m, 4H, H_{11}), 6.61 (s, 0.16H, *E,β*-H_9), 6.19 (s, 0.08H, *E,γ*-H_9), 6.02 – 5.94 (m, 0.99H, *Z,γ*-H_6), 5.94 – 5.79 (m, 2.23H, *E,γ*-H_6, *E,β*-H_6, *Z,γ*-

H_5, Z,β-H_6 and Z,β-H_5), 5.79 – 5.69 (m, 0.98H, E,γ-H_5, Z,γ-H_3 and Z,β-H_9), 5.69 – 5.65 (m, 0.29H, Z,β-H_2), 5.55 (s, 0.49H, Z,γ-H_9), 5.41 (dd, J = 11.1, 8.8 Hz, 0.29H, E,β-H_5), 5.34 (d, J = 7.0 Hz, 0.15H, E,β-H_2), 5.29 (d, J = 8.8 Hz, 0.08H, E,γ-H_3), 3.97 (s, 0.89H, Z,γ-H_8), 3.88 (s, 0.48H, Z,β-H_8), 3.86 (s, 0.14H, E,γ-H_8), 3.81 (s, 0.27H, E,β-H_8), 2.73 (t, J = 8.6 Hz, 0.51H, Z,γ-H_1), 2.59 (t, J = 8.7 Hz, 0.31H, Z,β-H_4), 2.49 – 2.43 (m, 0.52H, Z,γ-H_4), 2.43 – 2.37 (m, 1.03H, Z,γ-H_7), 2.32 – 2.26 (m, 0.10H, E,γ-H_4), 2.27 – 2.19 (m, 0.80H, Z,β-H_1 and Z,β-H_7), 2.18 – 2.11 (m, 0.26H, E,γ-H_1 and E,γ-H_7), 2.11 – 2.02 (m, 0.48H, E,β-H_1 and E,β-H_7), 1.72 (t, J = 8.8 Hz, 0.24H, Z,β-H_4), 1.42 (s, 1.36H, E,β-H_{16}), 1.41 (s, 0.70H, E,γ-H_{16}), 1.10 (s, 2.33H, Z,β-H_{16}), 1.03 (s, 4.32H, Z,γ-H_{16}). **^{13}C NMR** (126 MHz, $CDCl_3$, 219 K) δ 156.3 (Z,γ-C_{14}, E,β-C_{14} and E,γ-C_{14}), 156.1 (Z,β-C_{14}), 140.1 (Z,γ-C_{10}), 140.1 (Z,β-C_{10}), 139.8 (E,γ-C_{10}), 139.7 (E,β-C_{10}), 136.7 (Z,β-C_3), 136.1 (E,β-C_3), 135.1 (Z,γ-C_2), 134.5 (E,γ-C_2), (128.8–126.0 C_{13}, C_{12}, C_{11}, C_6, C_5 and Z,γ-C_3) 128.8, 128.7, 128.4, 128.3, 128.2, 128.0, 127.9, 127.5, 127.3, 127.2, 127.1, 127.0, 126.9, 126.8, 126.8, 126.4, 126.1, 126.0, 124.8 (Z,β-C_2), 122.3 (E,γ-C_3), 121.2 (E,β-C_2), 80.3 (C_{15}), 80.1 (C_{15}), 62.8 (E,γ-C_9), 62.7 (E,β-C_9), 62.6 (Z,β-C_9), 62.1 (Z,γ-C_9), 56.6 (Z,γ-C_8), 54.3 (Z,β-C_8), 54.2 (E,γ-C_8), 51.2 (E,β-C_8), 31.7 (Z,β-C_4), 30.4 (E,β-C_9), 29.9 (Z,γ-C_4), 29.6 (E,γ-C_4), 28.2 (C_{16}), 28.1 (C_{16}), 27.9 (C_{16}), 21.6 (Z,γ-C_1), 20.4 (Z,β-C_7 and E,γ-C_1), 20.2 (Z,γ-C_7), 20.0 (E,β-C_7), 19.9 (E,γ-C_7), 19.7 (Z,β-C_1), 19.3 (E,β-C_1). **HRMS ASAP** m/z = 426.2433 $[M+H]^+$, calculated for $C_{29}H_{32}NO_2^+$: 426.2432.

3. ^{1}H and ^{13}C NMR Spectroscopic Characterization

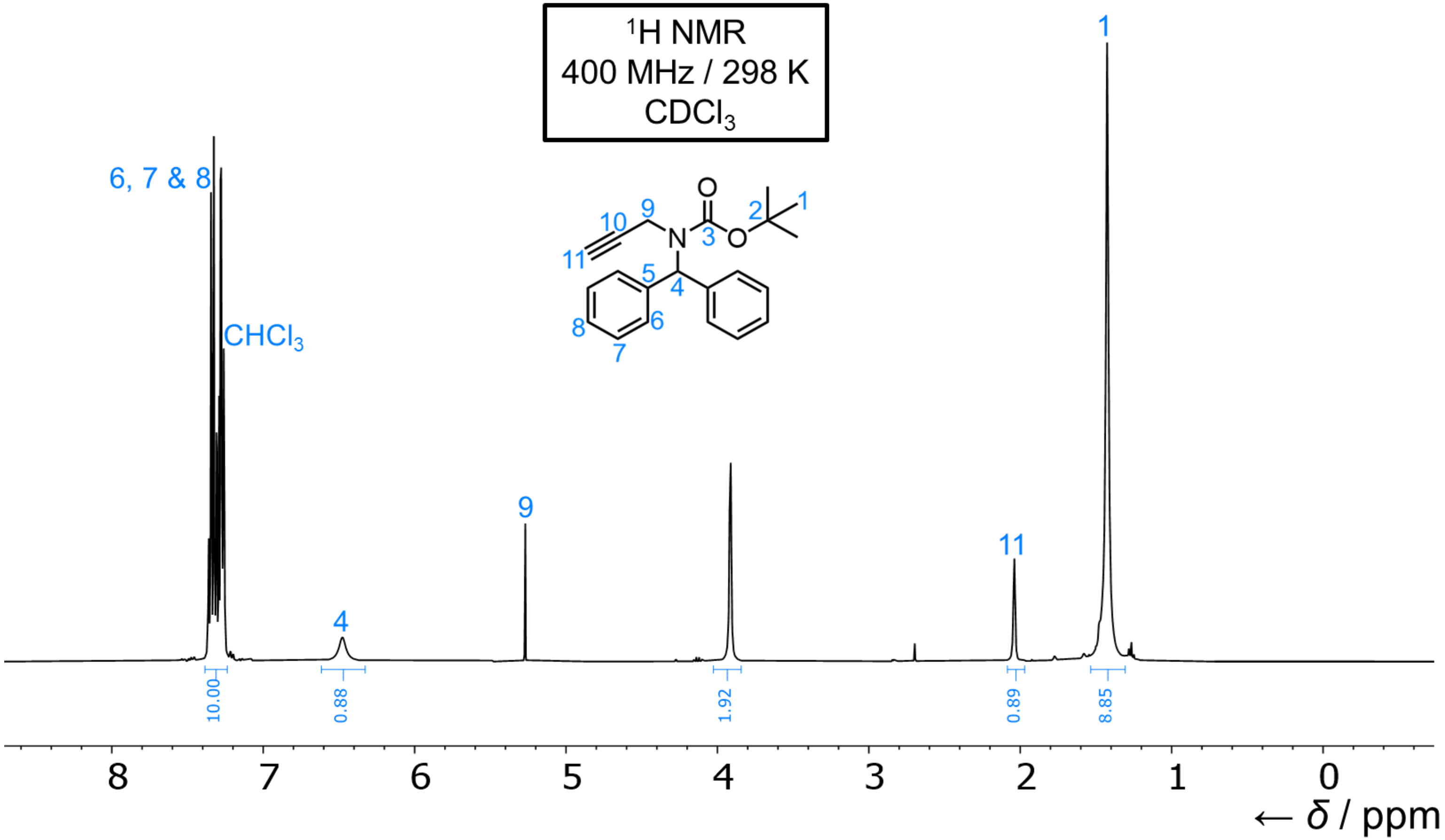

Figure S1. ^{1}H NMR spectrum of **4b**.

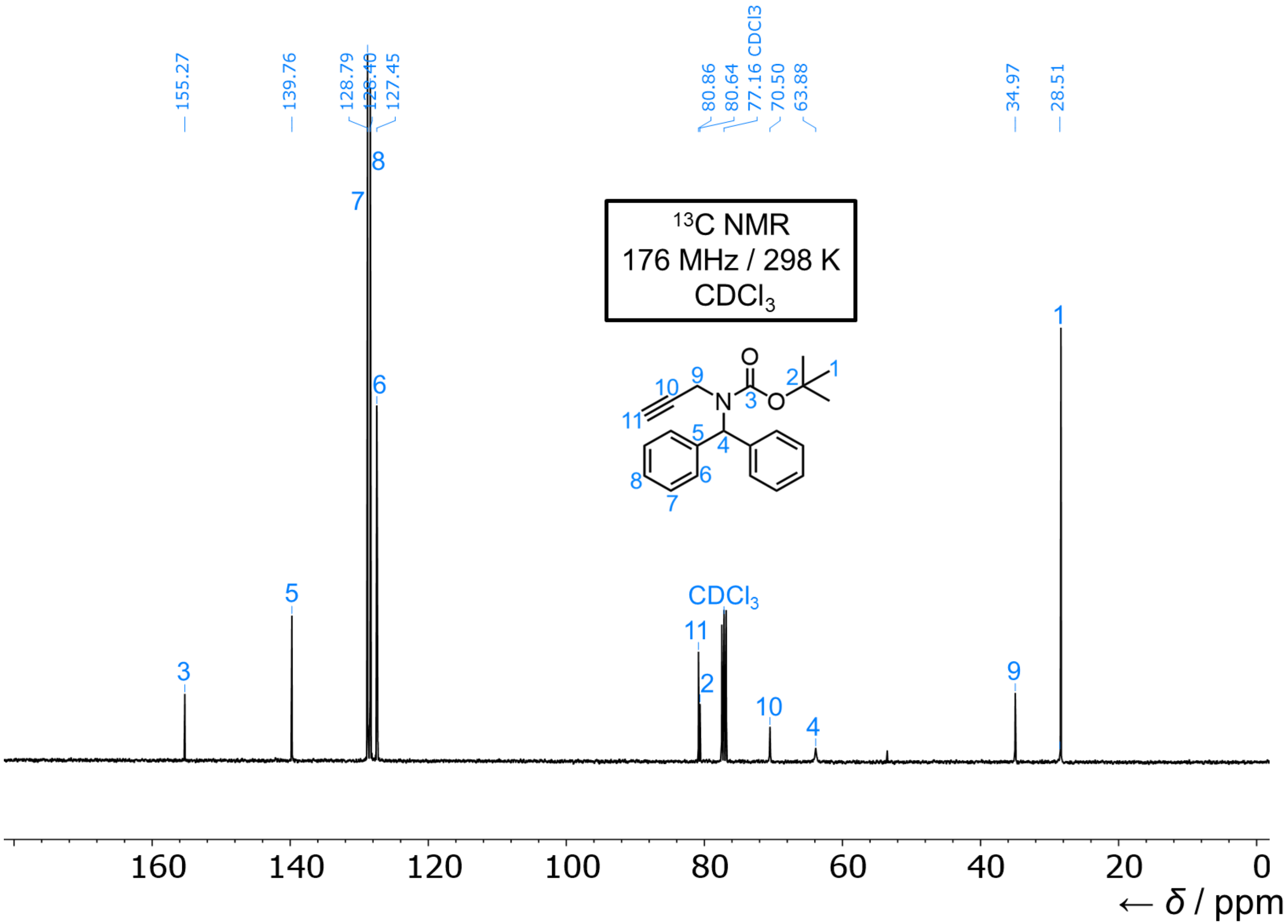

Figure S2. $^{13}C\{^{1}H\}$ NMR spectrum of **4b**.

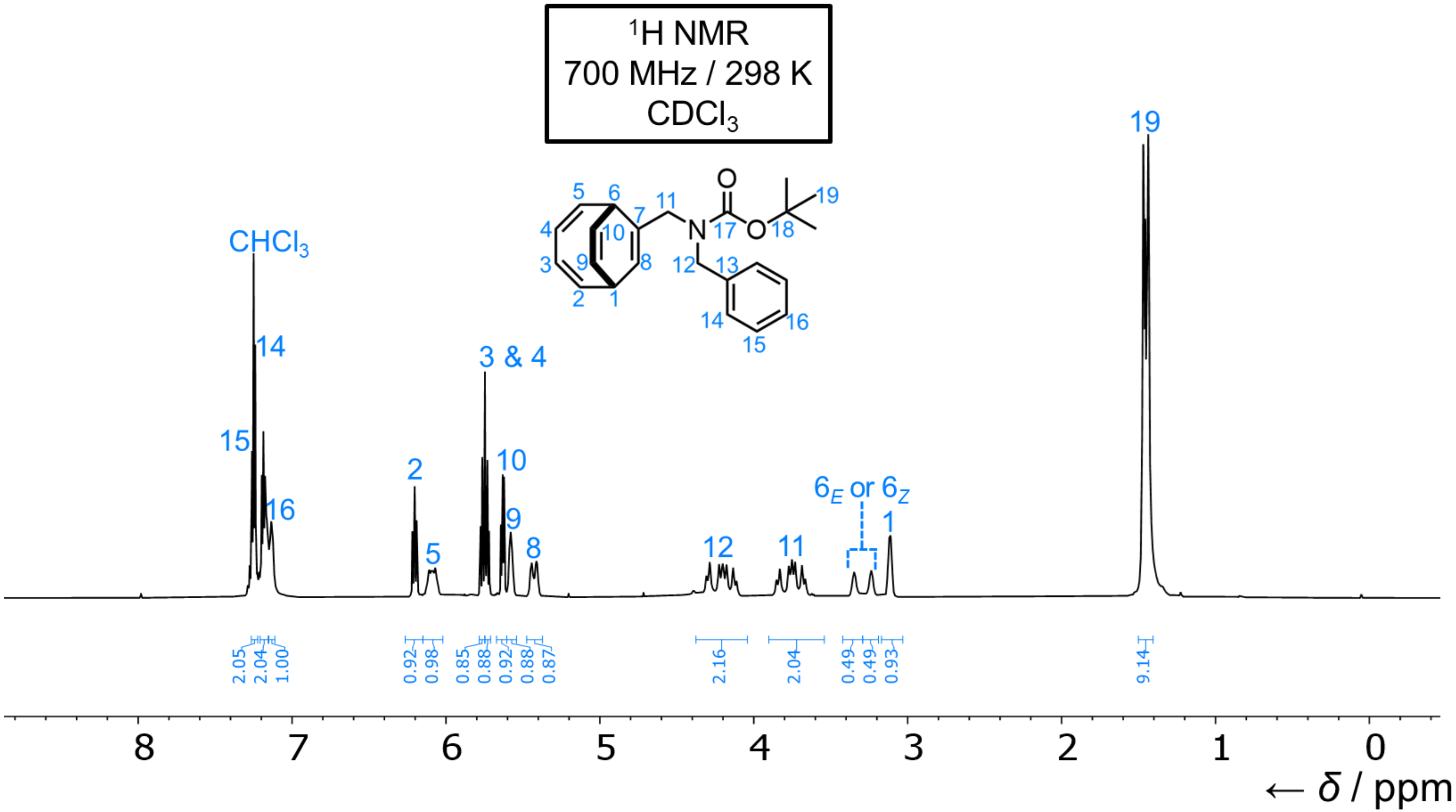

Figure S3. ^{1}H NMR spectrum of **5a**.

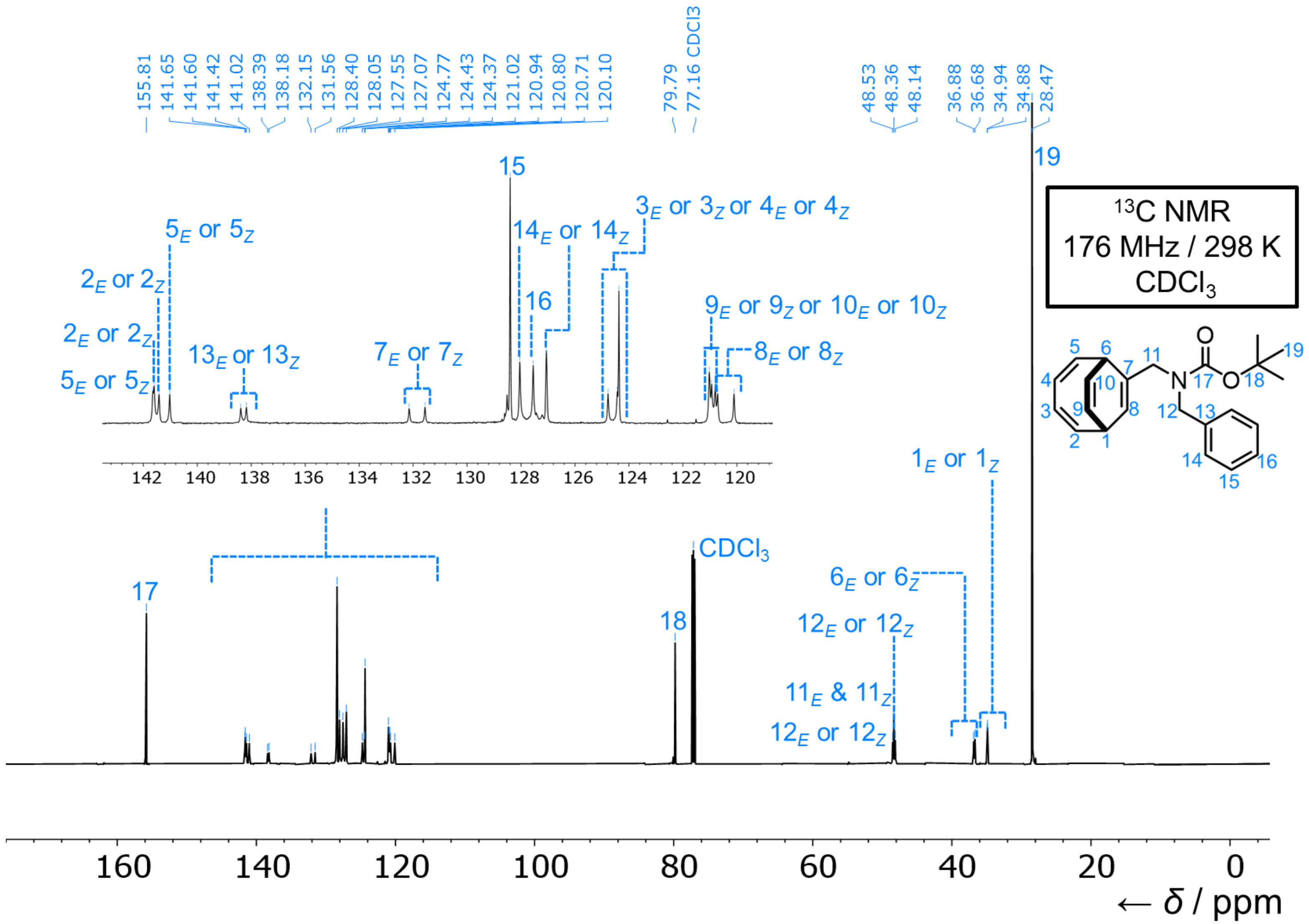

Figure S4. $^{13}C\{^1H\}$ NMR spectrum of **5a**.

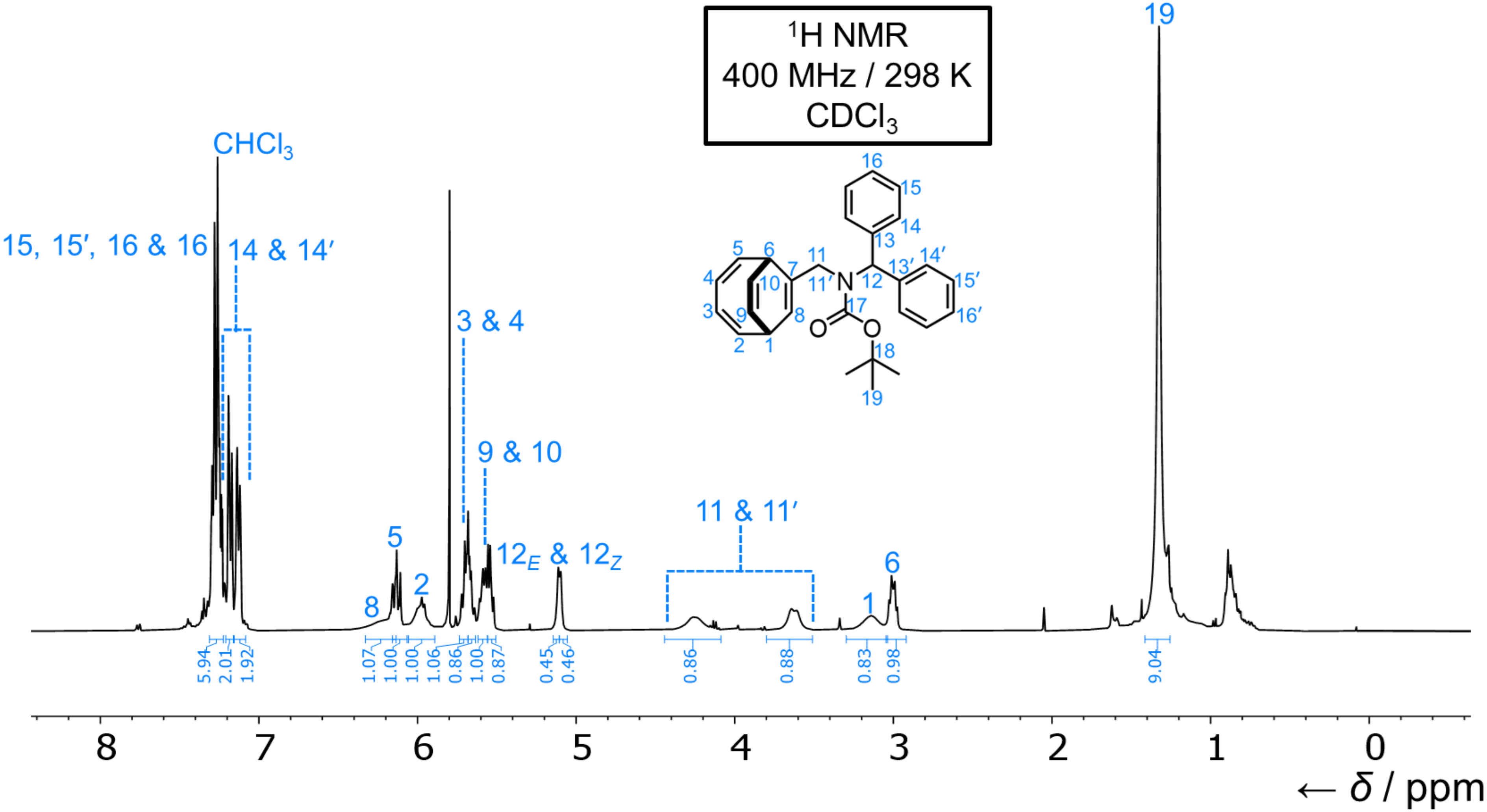

Figure S5. ^{1}H NMR spectrum of **5b**.

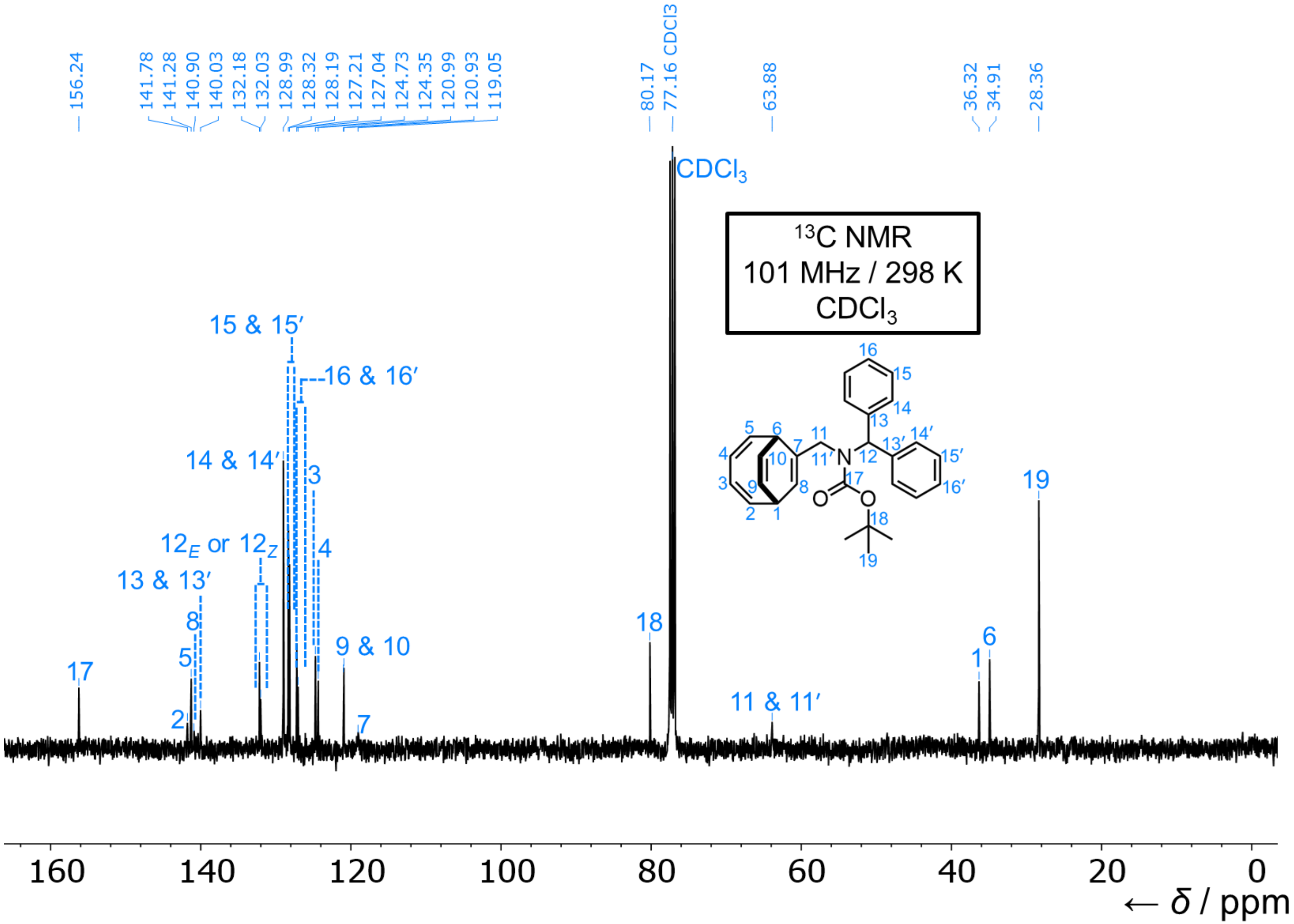

Figure S6. ^{13}C{^{1}H} NMR spectrum of **5b**.

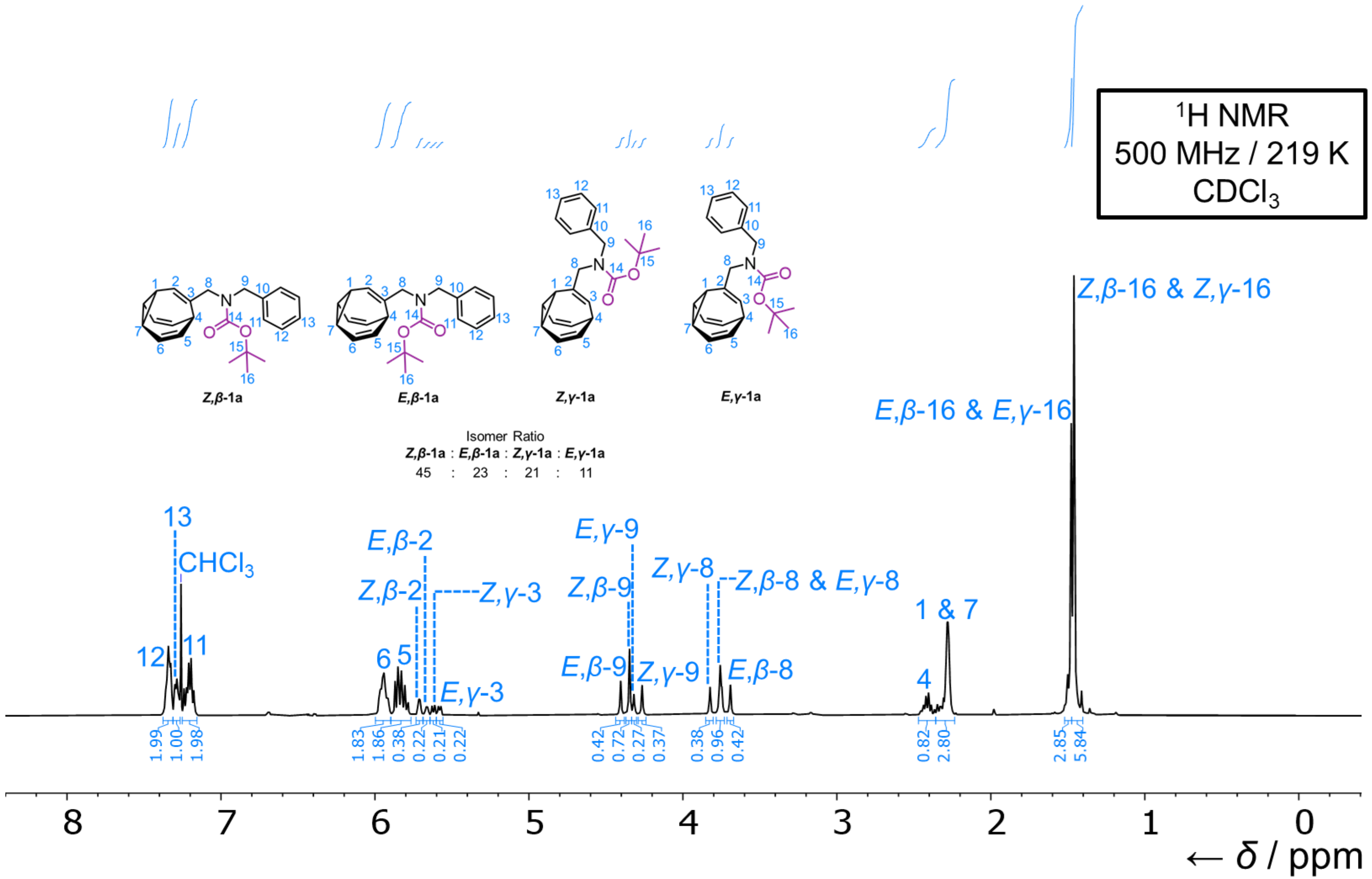

Figure S7. ¹H NMR spectrum of **1a**.

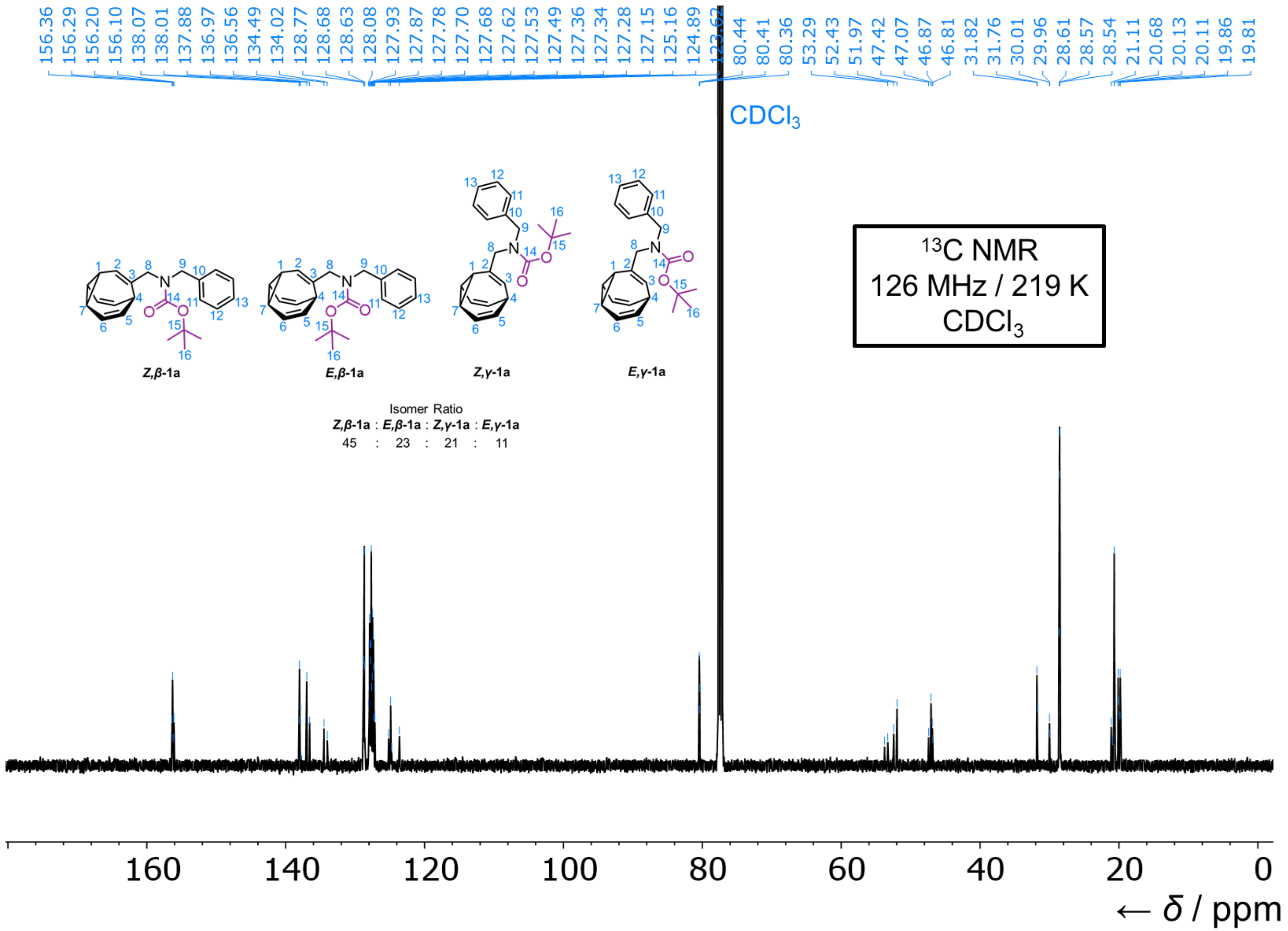

Figure S8. $^{13}C\{^{1}H\}$ NMR spectrum of **1a**.

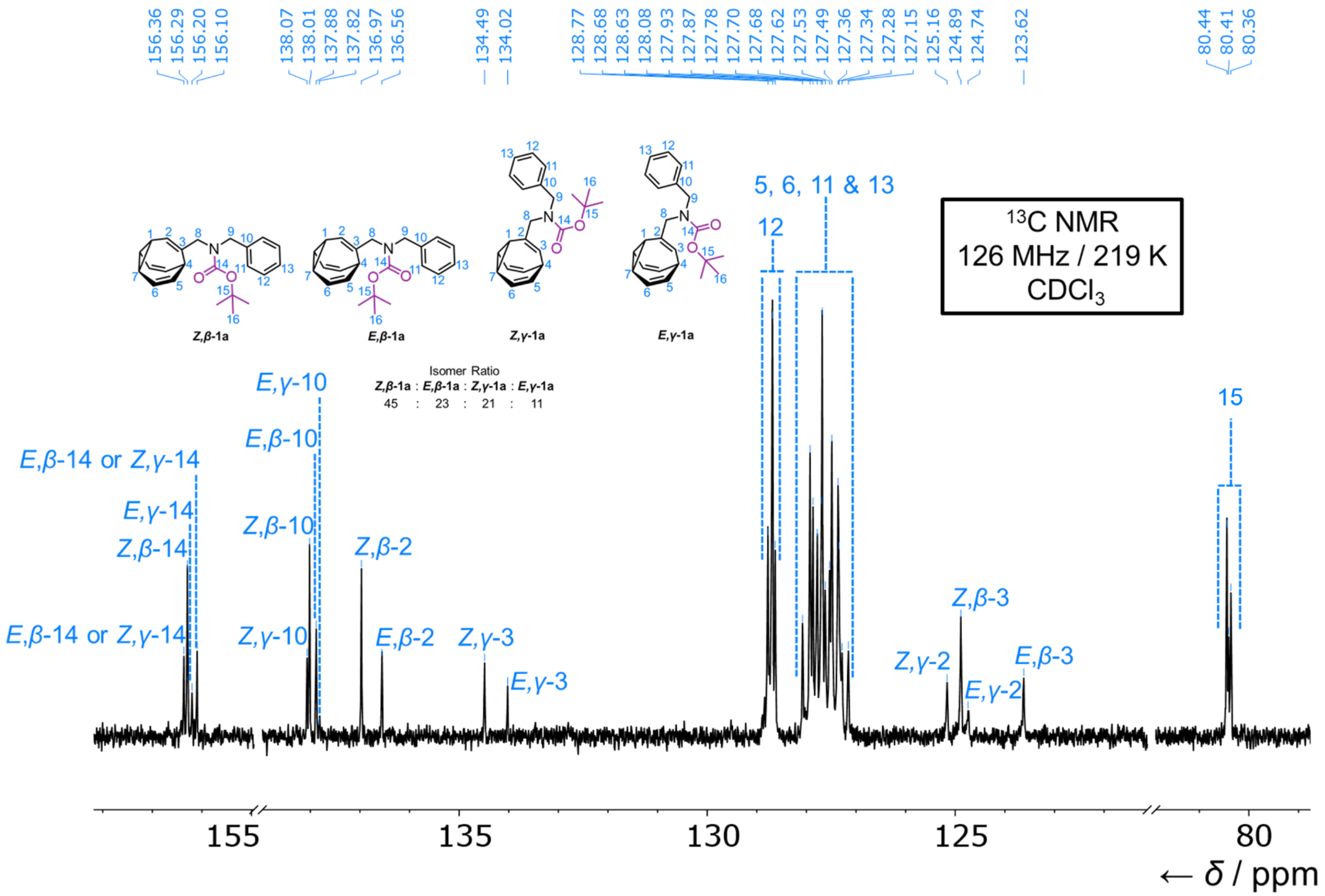

Figure S9. Partial $^{13}C\{^{1}H\}$ NMR spectrum of **1a** showing peak assignments.

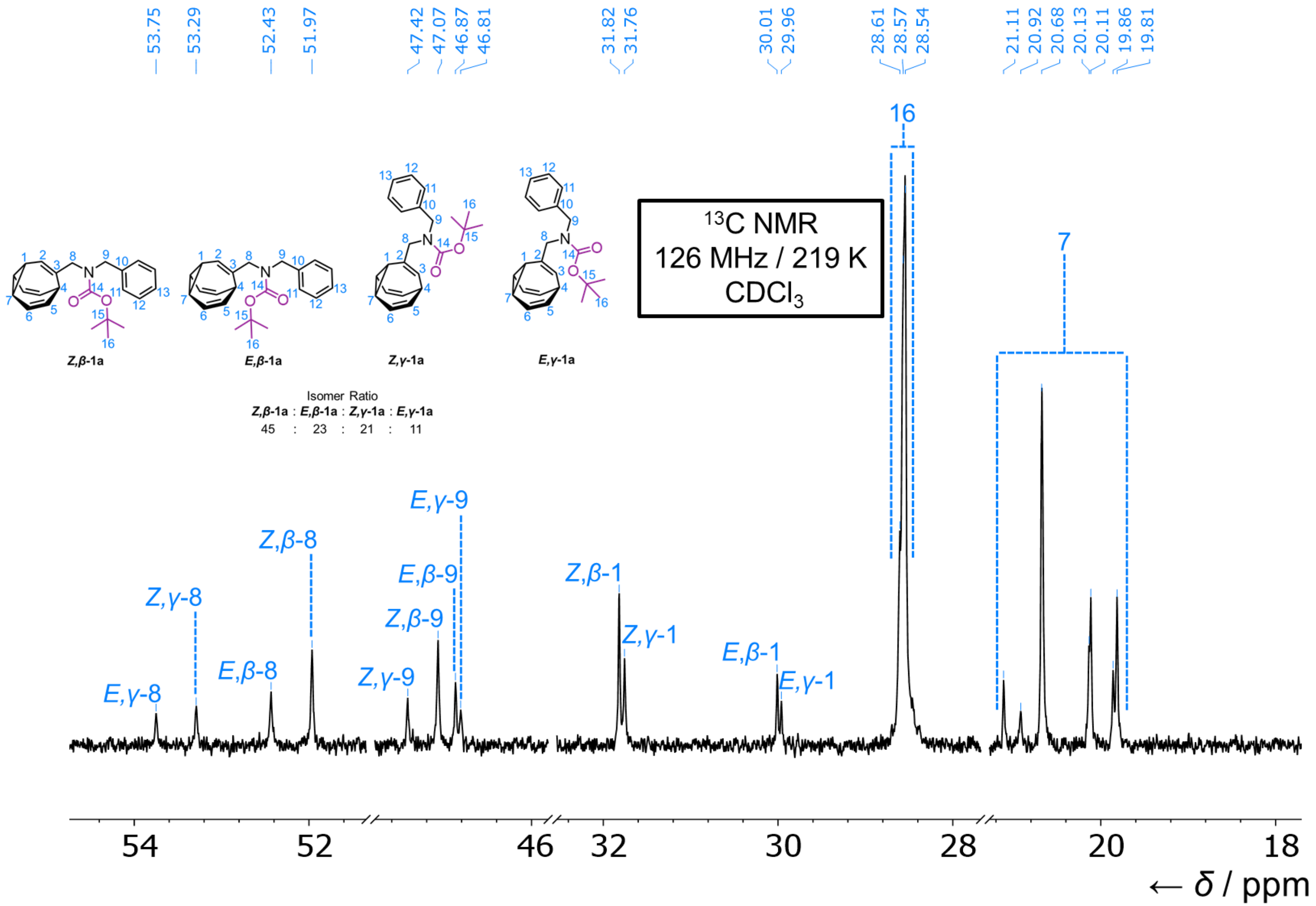

Figure S10. Partial ^{13}C{^{1}H} NMR spectrum of **1a** showing peak assignments.

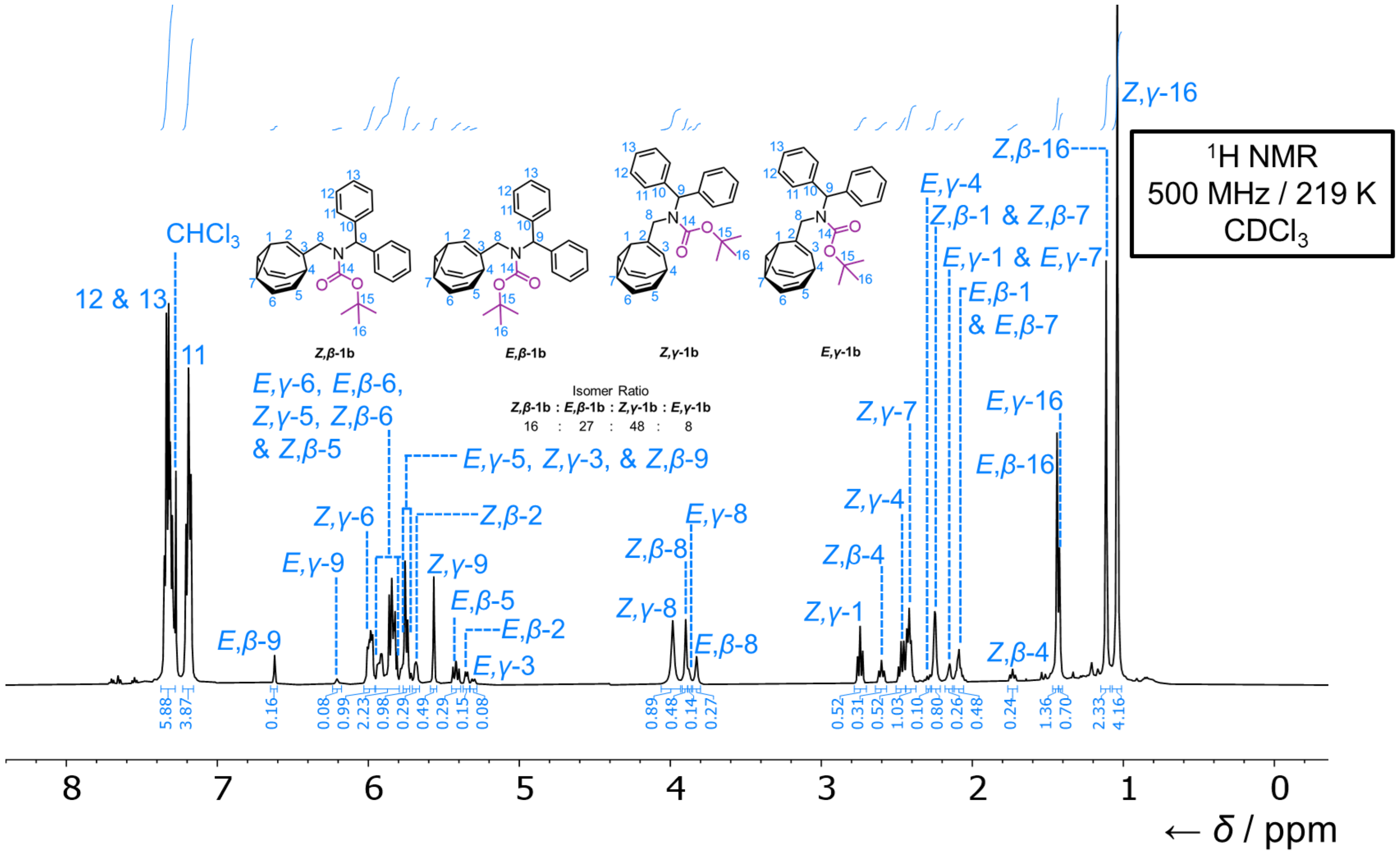

Figure S11. ^{1}H NMR spectrum of **1b**.

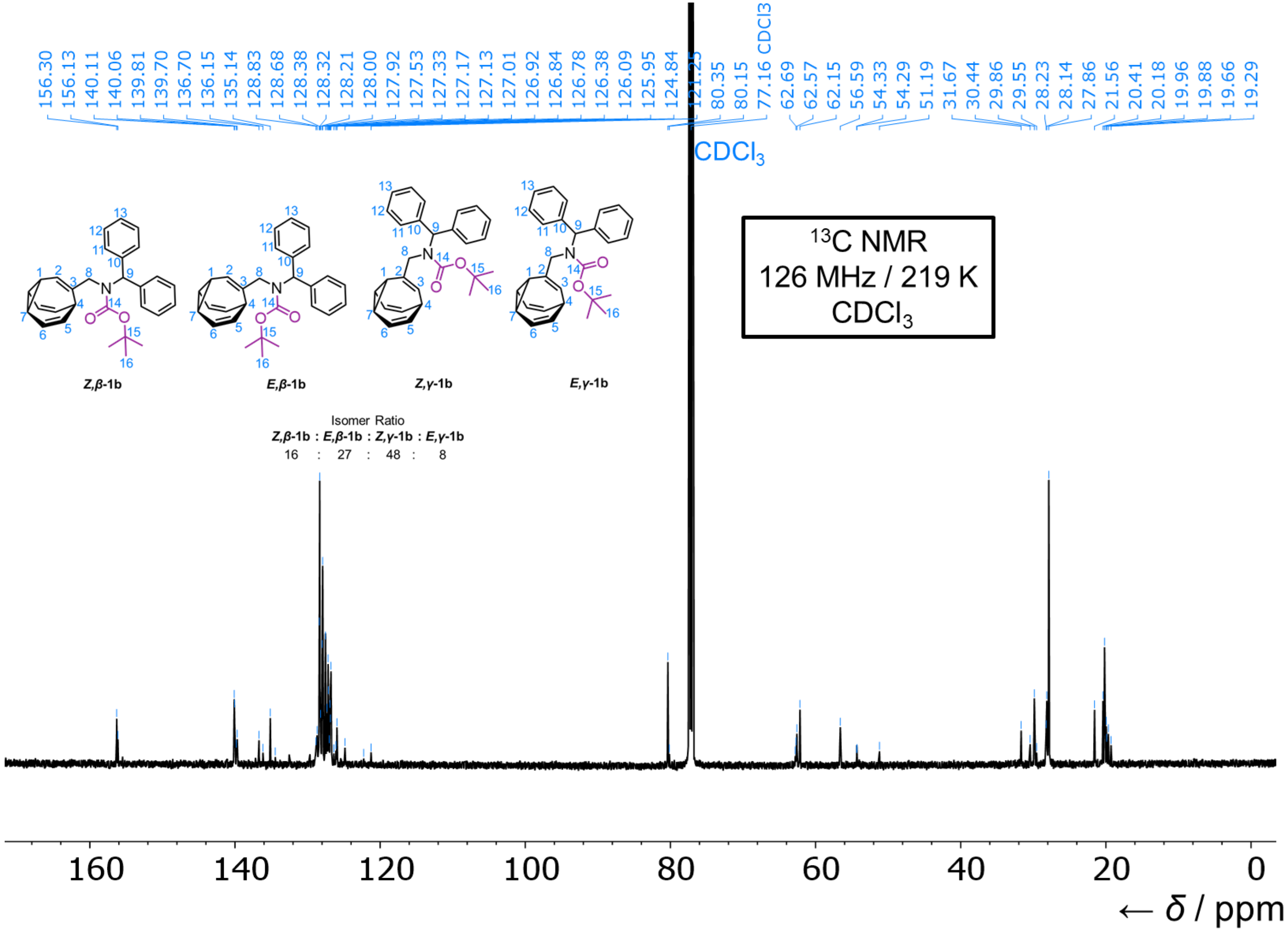

Figure S12. $^{13}C\{^{1}H\}$ NMR spectrum of **1b**.

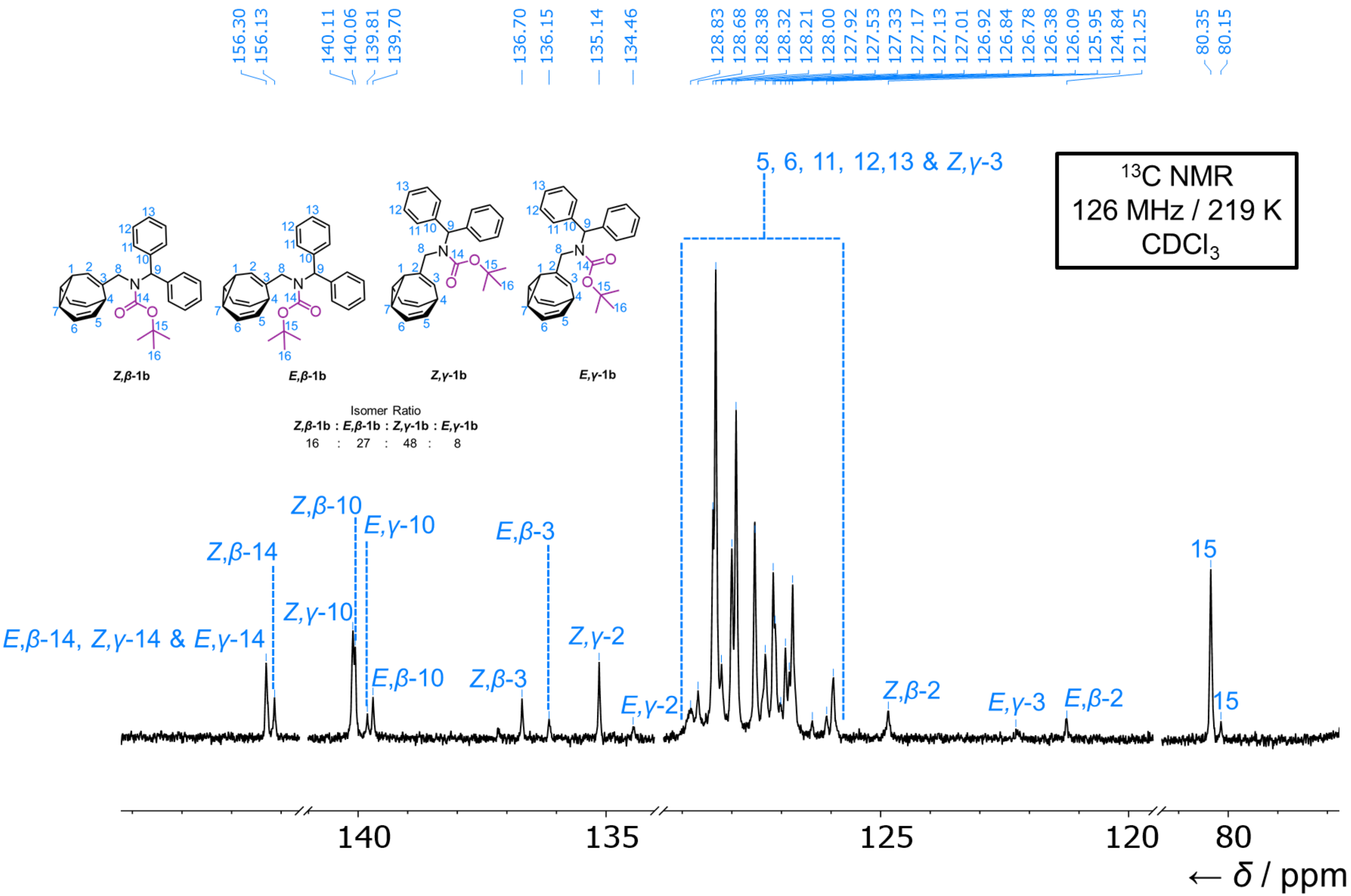

Figure S13. Partial $^{13}C\{^{1}H\}$ NMR spectrum of **1b** showing peak assignments.

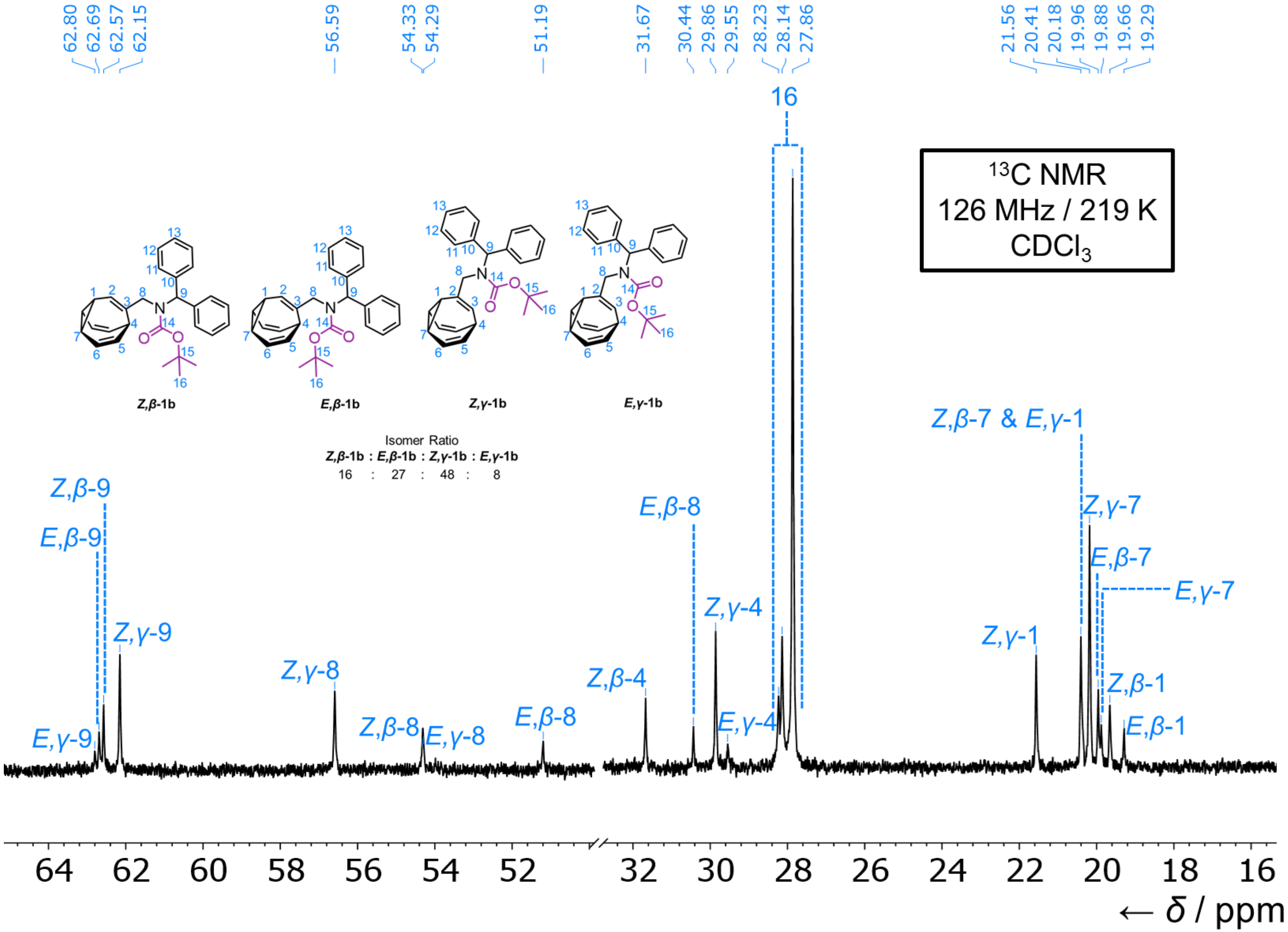

Figure S14. Partial $^{13}C\{^{1}H\}$ NMR spectrum of **1b** showing peak assignments.

4. Structural Assignment by 1D and 2D NMR

4.1. Benzhydryl Boc Bullvalene (1b)

The four most populated isomers of **1b** (Figure S15a) were distinguished through a combination of 1D and 2D NMR experiments (Figures S15–S17).

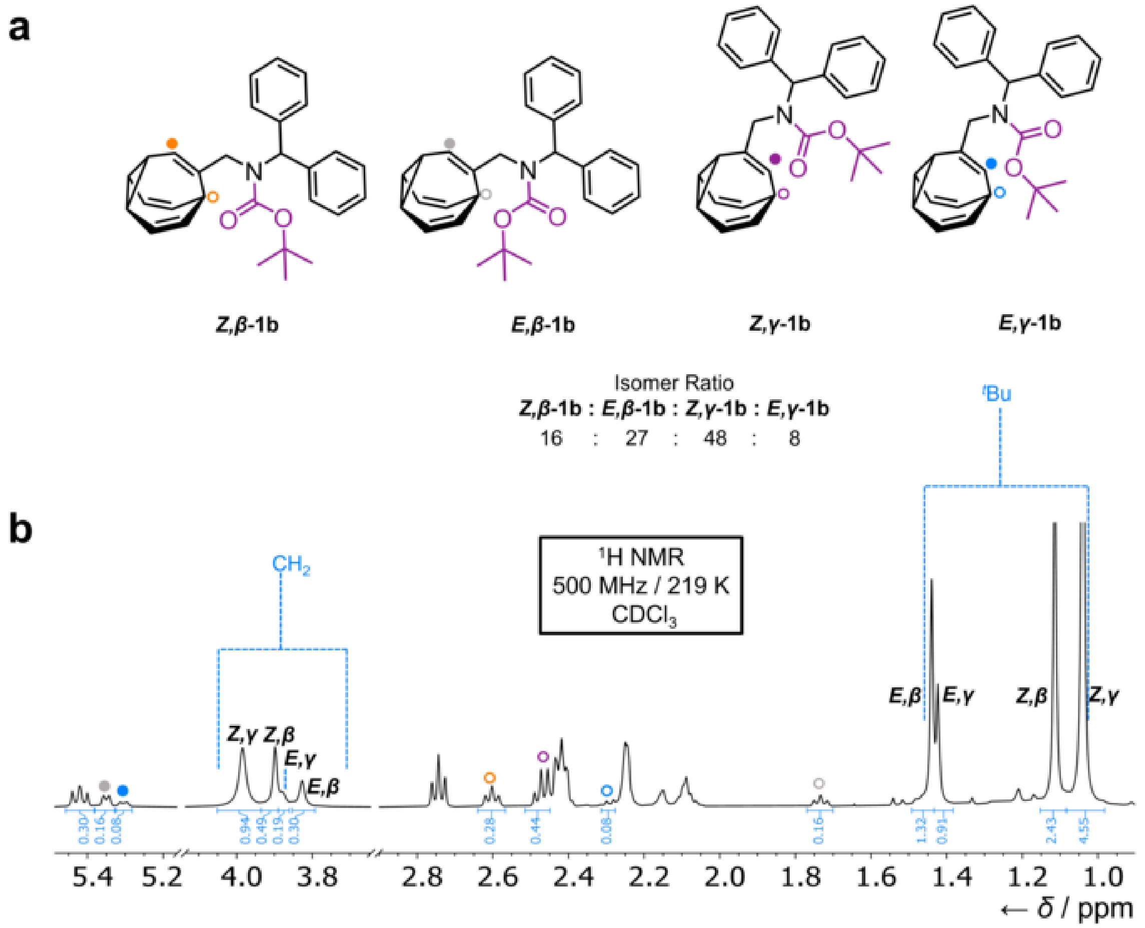

Figure S15. (a) Structural formulas of the **1b** isomers, which can be assigned in the (b) partial ^{1}H NMR spectrum.

The resonances corresponding to the methylene group (CH_2) on each isomer appear between 3.8–4.0 ppm in a ratio of 48:27:8:16. Based on the integration of the doublet at ~5.3 ppm (filled blue circle, Figure S15b), which is half that of the smallest methylene peak, this signal must correspond to a single methine group (a unique CH environment) of the least populated isomer. Considering the chemical shift of this peak and the symmetry of the bullvalene, the resonance

can be assigned to the alkene methine environment present on the same arm of the bullvalene as the carbamate substituent.

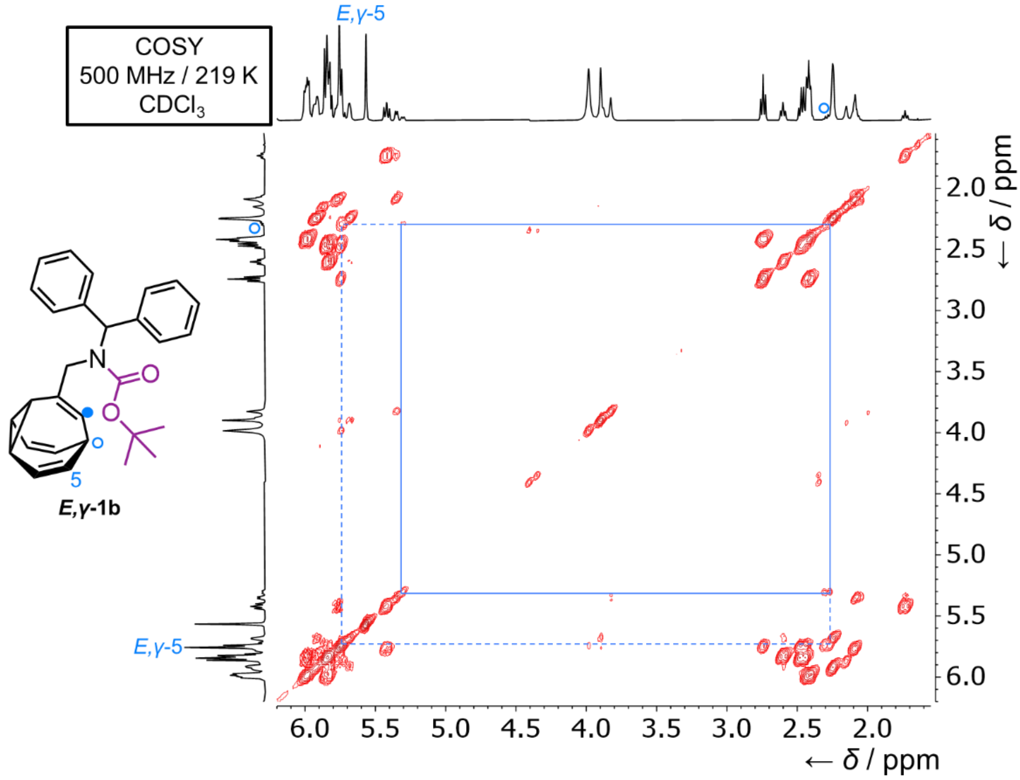

Figure S16. Partial ^{1}H–^{1}H COSY spectrum of the **1b** isomers.

The same signal shows a COSY correlation (solid blue lines, Figure S16) to a peak at ~2.3 ppm (hollow blue circle, Figure S16). This proton resonance at ~2.3 ppm is associated with a ^{13}C signal at ~30 ppm in the HSQC spectrum (solid blue line, Figure S17). Trivinyl bridgehead (α-position) ^{13}C resonances of bullvalenes typically appear at ~30 ppm, whereas cyclopropyl signals (δ-positions) appear in the 20–25 ppm region. The only other COSY correlation for the ~2.3 ppm proton environment is to another alkene CH position (***E,γ*-5**) (dashed blue line, Figure S16). An additional COSY cross peak to another signal in the sp^3 methine region of the spectrum would be expected if the signal were a cyclopropyl methine (δ-position), but no such cross peak is observed. These two observations are consistent with one another, and thus allow us to assign the ~2.3 ppm signal as an apical bridgehead (α-position) methine, rather than a

cyclopropyl signal (δ-position). Consequently, the minor isomer is assigned as a γ-substituted bullvalene as its alkene methine (β-position) correlates to the apical bridgehead (α-position).

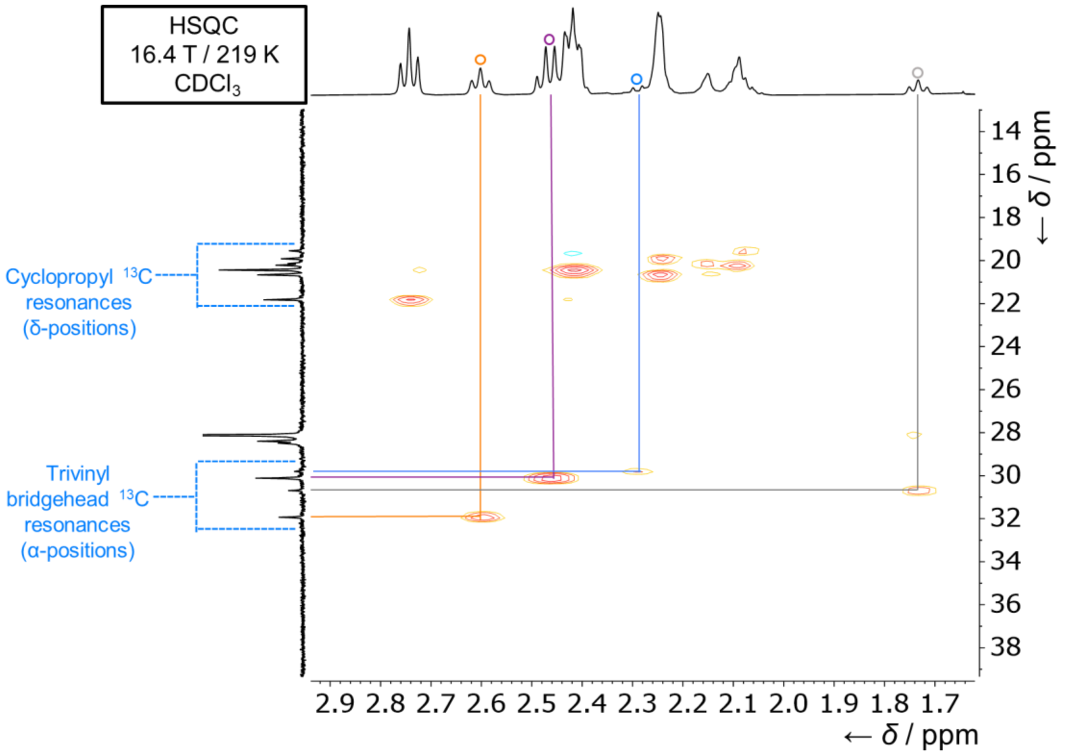

Figure S17. Partial ^{1}H–^{13}C HSQC NMR spectrum of the **1b** isomers.

To distinguish whether this minor isomer has *E*- or *Z*-carbamate stereochemistry, we performed a series of 1D NOESY experiments (Figure S18). The ^{t}Bu resonances in the 1.0–1.5 ppm region, were excited to look for through-space close contacts. Exciting the two most intense ^{t}Bu signals at 1.05 ppm (Figure S18c) and 1.12 ppm (Figure S18b) while allowing a mixing time of 250 ms, causes magnetization to be transferred to aromatic signals above 7.0 ppm. No such interaction is observed (Figure S18a) when exciting the overlapping ^{t}Bu signals at ~1.43 ppm (the two least intense peaks). Consequently, the two major isomers are assigned as having *Z* stereochemistry, which brings the ^{t}Bu into closer contact with the Ph groups and gives the observed nuclear Overhauser effect. Conversely, the two minor isomers have *E* stereochemistry.

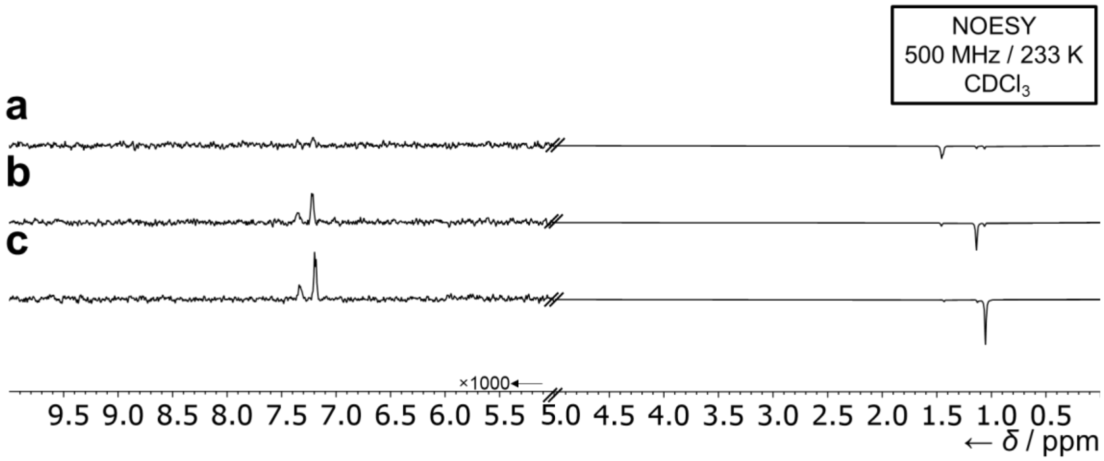

Figure S18. A series of selective refocussing partial NOESY spectra acquired using 250 ms mixing times after refocussing at frequencies corresponding to (a) 1.43 ppm, (b) 1.11 ppm and (c) 1.04 ppm. Note that the intensity of the spectra above 5 ppm has been scaled by a factor of 1000 to show the weak nuclear Overhauser effect between the Ph group and ^{t}Bu signals of the *Z*-isomers (i.e., spectra b and c), which is absent for the *E*-isomers (spectrum a). Positive peaks indicate spatial proximity. Negative peaks indicate resonances that have been selected and/or are in chemical exchange.

Overall, therefore, the least populated isomer is assigned as being ***E*,*γ*-1b**. Using the same assignment procedure for the other isomers, with the additional information about which isomers are in direct exchange provided by EXSY NMR (Figure 3c of the main text) allows identification of the other three isomers in the same manner.

4.2. Benzyl Boc Bullvalene (1a)

The isomers of **1a** (Figure S15a) were assigned in a similar manner to **1b** using 2D NMR techniques at ~219 K (Figures S15 to S18).

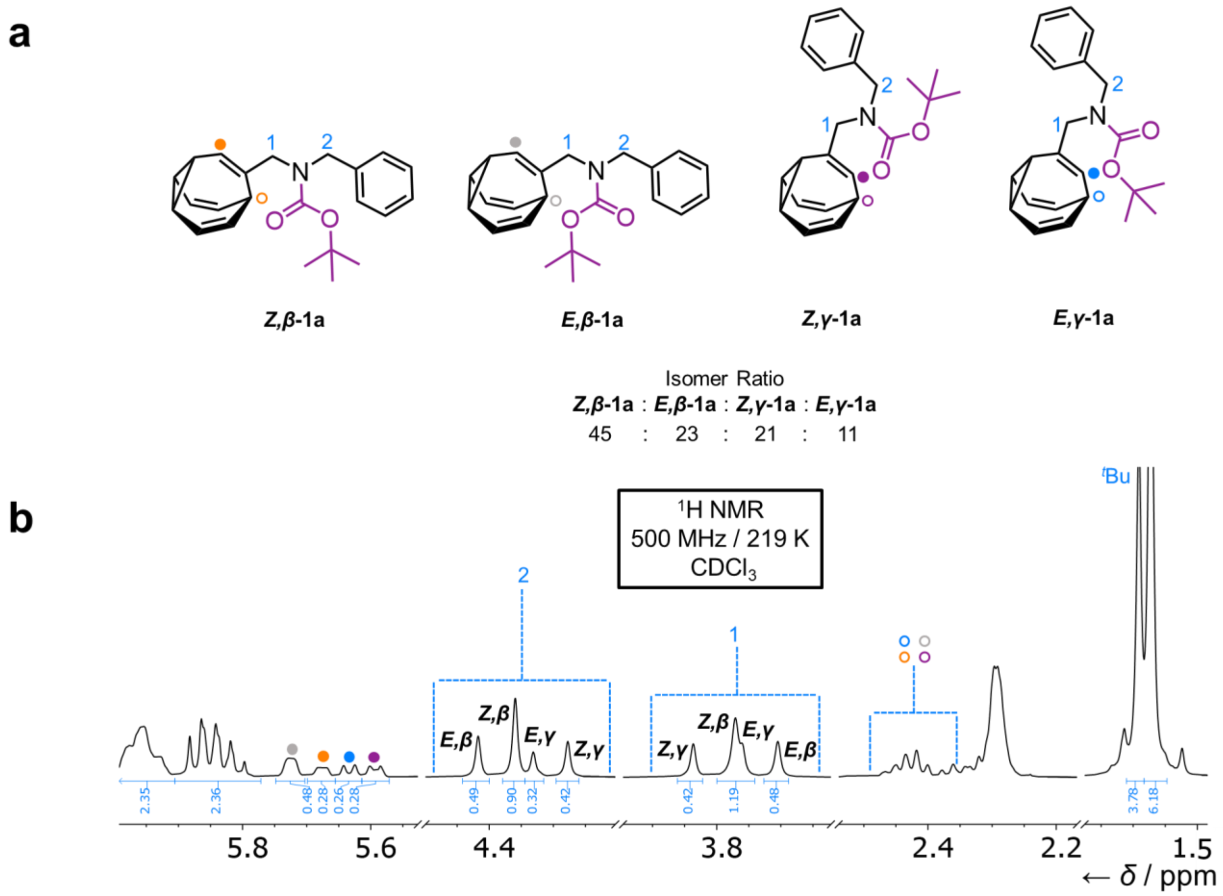

Figure S19. (a) Structural formulas of the **1a** isomers, which can be assigned in (b) the partial ^{1}H NMR spectrum.

Four signals appear at 5.55–5.75 ppm (Figure S19b) that each correspond to the single alkene methine present on the same arm of the bullvalene as the carbamate substituent. Each of these correlates to a H1 methylene signal by ^{1}H–^{1}H COSY NMR (Figure S20). The two highest field alkene methines (blue and purple filled circles) appear as doublets with *J* coupling values of ~8 Hz, whereas the lower field methines (orange and grey filled circles) appear as broad singlets, likely because of them experiencing small *J* coupling (<6 Hz) that is not resolved in the spectrum. The two doublets at high field couple to (blue and purple lines, Figure S20) a

cluster of signals just above 2.4 ppm. These proton resonances can be assigned (colored hollow circles) as an apical bridgehead (α-positions) by analysis of ^{1}H-^{13}C HSQC (black lines, Figure S21). All four of the apical ^{13}C signals >30 ppm (α-positions) correlate to proton resonances at 2.35 ppm and above, whereas the cyclopropyl (δ-positions) ^{13}C signals (at ~20 ppm) correlate to the resonances below 2.35 ppm. Therefore, the alkene methine doublets with *J* coupling of 8 Hz (blue and purple filled circles) can both be attributed to *γ*-substituted bullvalene isomers as they show coupling to neighboring bridgehead protons (α-positions), rather than to cyclopropyl signals (δ-positions) as would be expected for *β*-substituted bullvalene isomers. Indeed, the other two alkene methine signals (orange and grey filled circles) show COSY correlations (Figure 20) with the cluster of overlapping cyclopropyl methine resonances at 2.3 ppm, which is consistent with them being part of *β*-substituted bullvalene isomers.

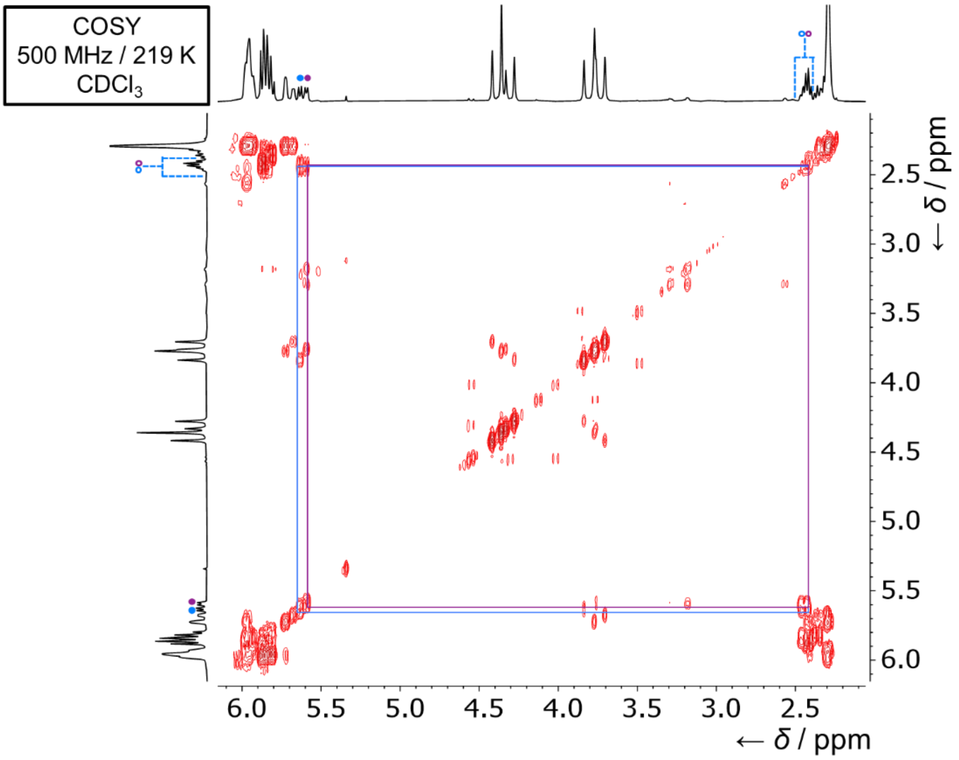

Figure S20. Partial ^{1}H–^{1}H COSY spectrum of the **1a** isomers.

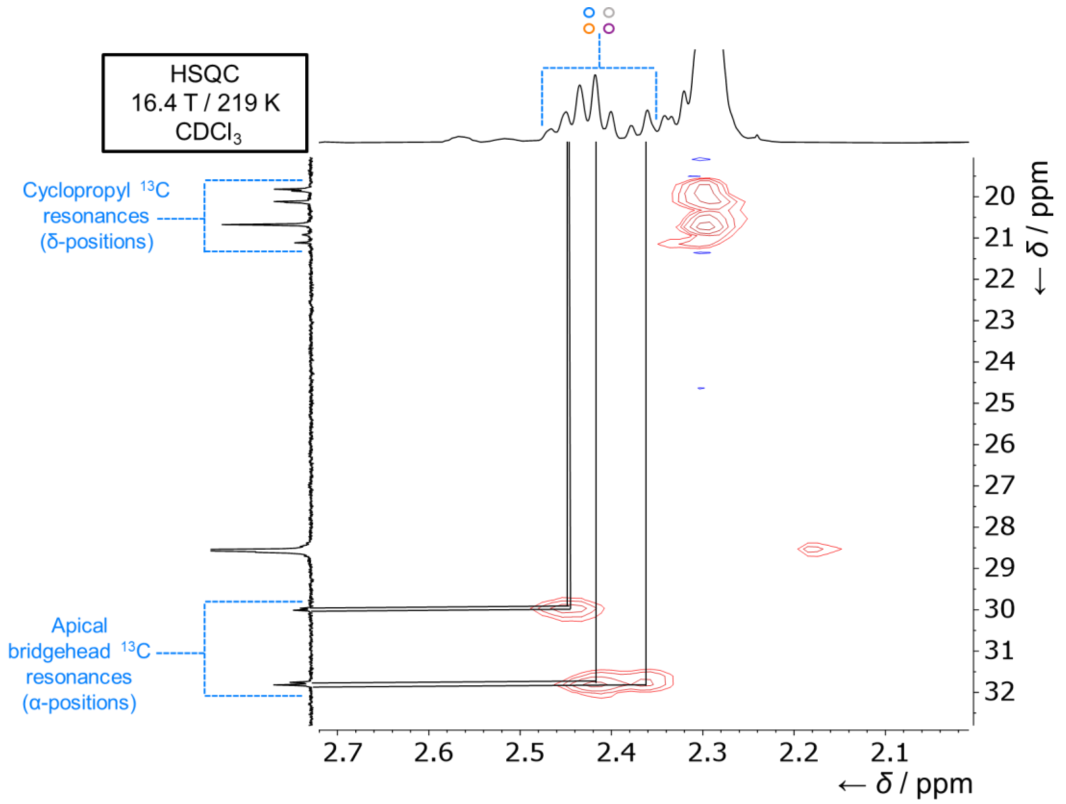

Figure S21. Partial ^{1}H–^{13}C HSQC NMR of the **1a** isomers.

Analysis of a ^{1}H EASY–ROESY NMR spectrum (red lines, Figure S22) allows us to distinguish the *E*- and *Z*-carbamate isomers. Of the four peaks in the H2 region (~4.4 ppm), only two show substantial through-space correlation to the ^{t}Bu signals. The H2 position would only be expected to be close in space to the ^{t}Bu group in the *Z*-isomer, i.e., when the benzyl and O^{t}Bu groups are both on the same side of the carbamate C–N bond. Therefore, we can assign the two signals as arising from the *Z*-isomer, as shown in Figure S19a. Consistently with this assignment, we also observe through-space correlation in the H1 region (~3.8 ppm) between the highest-field resonance and a ^{t}Bu signal. The H1 position comes into close contact with to the ^{t}Bu when they are both on the same side of the carbamate C–N bond in the *E*-isomer. This resonance can therefore be assigned to the *E* isomer, which matches the assignment made using the H2 region.

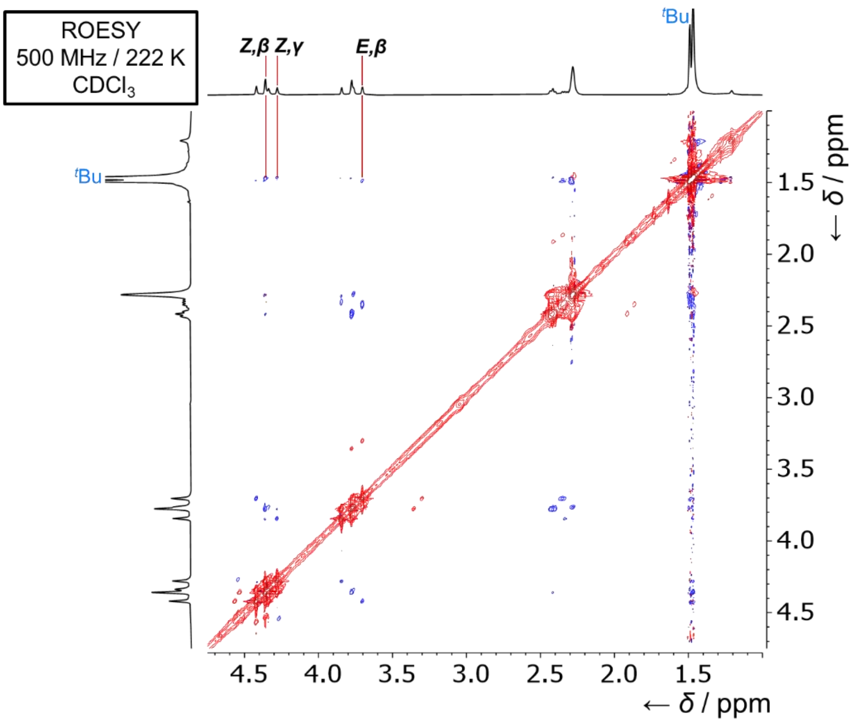

Figure S22. Partial ^{1}H–^{1}H EASY-ROESY NMR spectra of the **1a** isomers acquired using 250 ms mixing time. Blue off-diagonal peaks indicate spatial proximity, red off-diagonal peaks indicate exchange, and blue and red peaks indicate COSY-like peaks.

Line-shape analysis was performed using WinDNMR 7.1. The signal at ~4.35 ppm was simulated as a 2-spin system using the parameters in Table S1. The population of ***E*-1a** was set to 55% based on the experimentally measured *E*-*Z* ratio at 282 K, which was the closest temperature at which the two signals could be resolved. Note that the *E*-*Z* ratio changes with temperature as the available thermal energy alters the Boltzmann distribution of isomers, so this 55:45 ratio is slightly different to the 66:33 ratio observed at 219 K (Figure 2b of the main text).

Table S1. Parameters for line-shape analysis.

Isomer	δ / ppm	ν / Hz	Population / %	Width at half height / Hz
***Z*-1a**	4.417	2208	45	4
***E*-1a**	4.343	2172	55	4

The line shape best matched the experimental data using combined rates for the forward and backward reaction k_{EZ}+k_{ZE} of 139 Hz. Based on the approximated ratio *E*-*Z* ratio of 55:45 at this temperature, k_{EZ} = 62.5 Hz and k_{ZE} = 76.5 Hz. Using the Eyring equation, the transition state energy relative to ***E*-1a** (Table S1) was then calculated based on this rate, k_{EZ}, at 308 K.

5. NOESY NMR Analysis

Analysis of **1b** by ^1H–^1H NOESY NMR shows a through-space correlation between the resonance at 2.73 ppm (filled purple square, Figure S23) and the resonance at 7.22–7.14 ppm (purple hollow square, Figure S23). These resonances correspond to the δ-position vicinal to the substitution on the bullvalene, and the *ortho*-aromatic proton, both on the **Z,γ**-isomer. This correlation supports the interaction occurring between the phenyl ring and bullvalene proton identified in the non-covalent interaction (NCI) calculations (Figures S26–S29).

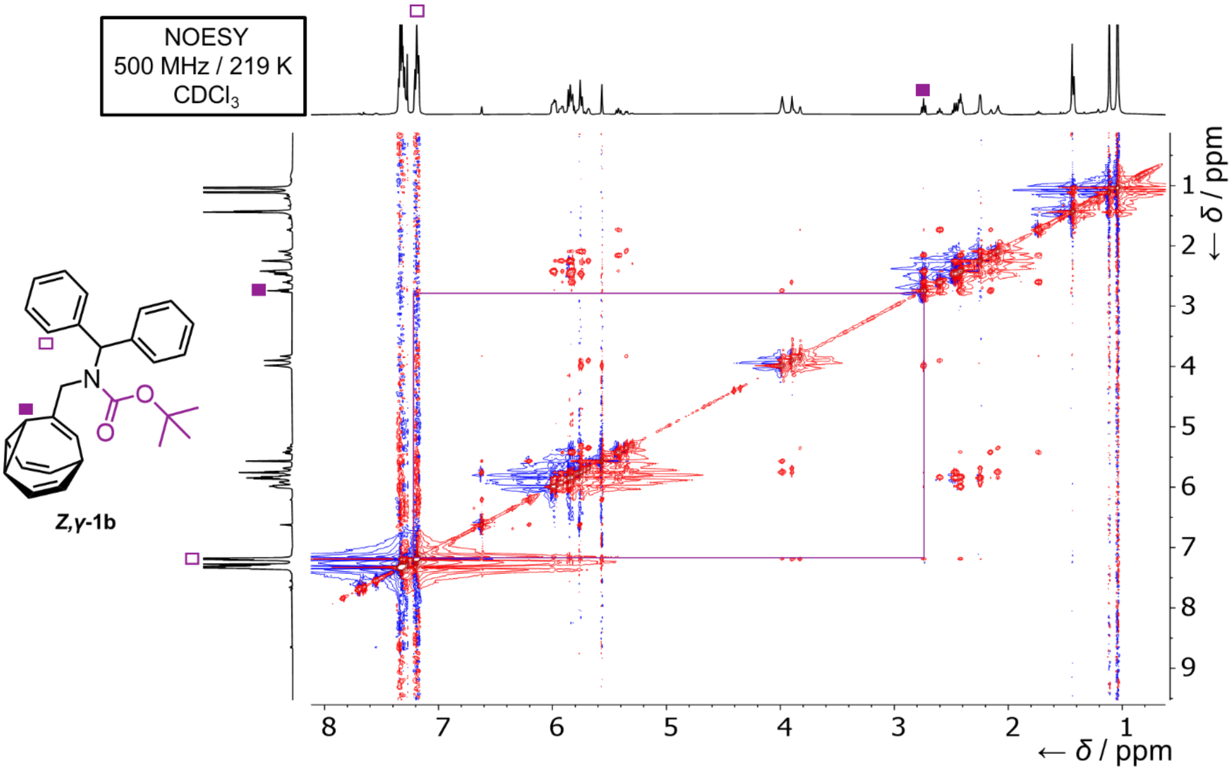

Figure S23. Partial ^1H–^1H NOESY NMR spectra of the **1a** isomers. Red off-diagonal peaks indicate spatial proximity, blue off-diagonal peaks indicate exchange, and blue and red peaks indicate COSY-like peaks.

6. X-Ray Crystallography

Crystals of ***E*,γ-1a** suitable for X-ray diffraction were grown by slow cooling of a MeOH solution of **1a** over 48 h from 60 °C to rt.

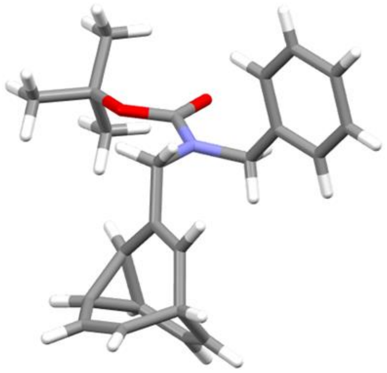

Figure S24. Solid-state structure of ***E*,γ-1a**

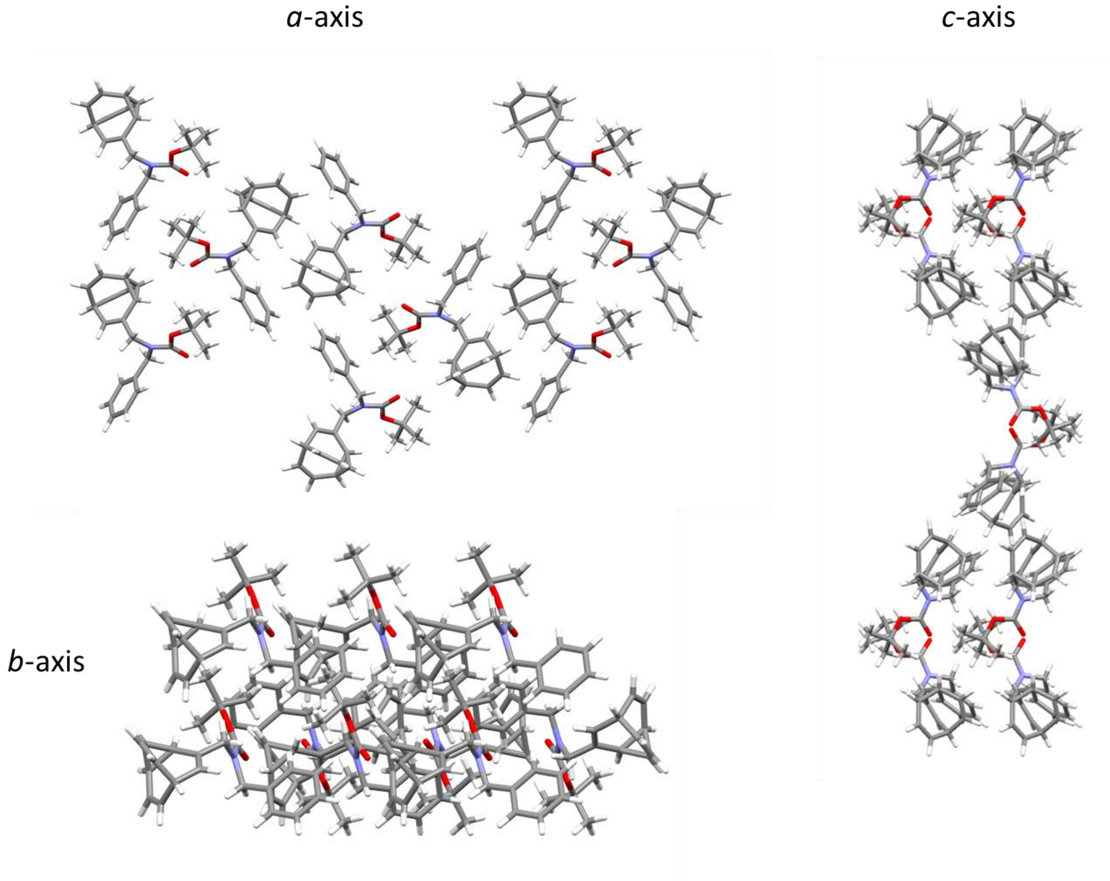

Figure S25. Solid-state superstructure of ***E*,γ-1a** viewed along the three unit cell axes.

Table S2. Crystal data and structure refinement for ***E,y*-1a**.

	***E,y*-1a**
CCDC number	2294194
Empirical formula	$C_{23}H_{27}NO_2$
Formula weight	349.45
Temperature/K	120.00
Crystal system	monoclinic
Space group	$P2_1/c$
a/Å	6.0431(2)
b/Å	26.5905(8)
c/Å	11.6793(3)
α/°	90
β/°	101.7723(10)
γ/°	90
Volume/Å^3	1837.26(9)
Z	4
ρ_{calc}g/cm^3	1.263
μ/mm^{-1}	0.624
F(000)	752.0
2Θ range for data collection/°	6.648 to 144.954
Index ranges	$-7 \leq h \leq 7$, $-32 \leq k \leq 32$, $-14 \leq l \leq 14$
Reflections collected	23653
Independent reflections	3612 [R_{int} = 0.0267, R_{sigma} = 0.0196]
Data/restraints/parameters	3612/0/343
Goodness-of-fit on F^2	1.025
Final R indexes [I>=2σ (I)]	R_1 = 0.0333, wR_2 = 0.0763
Final R indexes [all data]	R_1 = 0.0343, wR_2 = 0.0770
Largest diff. peak/hole / e Å^{-3}	0.25/-0.17

7. Computational Details

7.1. Conformer Generation

All initial sets of Cartesian coordinates sampling the shapeshifting and conformational isomers of **1a** and **1b** were generated *via* the in-house-developed *bullviso*[20a] code. *bullviso* is publicly available under the GNU Public License (GPLv3) on GitLab. The *α*-, *β*-, *γ*-, and *δ*-constitutional isomers of **1a** and **1b** were generated systematically/exhaustively using *bullviso*; the configurational and conformational isomers were generated according to the experimental-torsion distance geometry (ETDG) with 'basic knowledge' (+K) embedding approach (ETKDGv3)[20b,c] as implemented in RDKit.[20d,e] An root mean square deviation (RMSD) filter with a threshold of 0.5 Å was used to prune the initial set of configurational and conformational isomers. All configurational and conformational isomers passing the RMSD filter were subsequently (pre-)optimized at the GFN2-xTB[20f,g] (extended tight binding; xTB) level of theory using *xtb* (v6.4.1).[20h] An SCF convergence criterion of 1.0×10^{-6} a.u. was used with convergence criteria of 5.0×10^{-6} and 1.0×10^{-3} a.u. for the energy change and gradient, respectively, in all geometry optimizations. All unique configurational and conformational isomers verified at the GFN2-xTB level of theory were progressed to density functional theory (DFT) geometry optimization.

7.2. DFT Geometry Optimization

All DFT geometry optimizations and energy evaluations of **1a** and **1b** were carried out at the PBE0-D3 level of theory (*i.e.* with the PBE0[20i-k] density functional of Adamo and Barone coupled with the D3[20l] dispersion correction of Grimme *et al.*) using ORCA (v5.0.2).[20m-o] All calculations were carried out under the resolution-of-identity (RI) approximation for the Coulomb integrals (RIJONX). A tightened SCF convergence criterion of 1.0×10^{-9} a.u. was used in all calculations; convergence criteria of 2.0×10^{-7} and $3.0{\times}10^{-5}$ a.u. were used for the energy change and gradient, respectively, in all geometry optimizations. The def2-SV(P)[20p] basis set of Weigend and Ahlrichs was coupled with the def2/J[20q] auxilliary basis set; the two were used together throughout. The proper convergence of all geometry optimizations to real minima was verified *via* vibrational frequency inspection.

Table S3. Summary of electronic energies, E_{SCF}, Gibbs energies, G_{219K}, and relative Gibbs energies, ΔG_{219K}, for the isomers of **1a**. All values of ΔG_{219K} are tabulated relative to the lowest-energy isomer of **1a**. All values are given as evaluated at the PBE0-D3/def2-SV(P) level of theory.

Isomer	E_{SCF} / Hartree	G_{219K} / Hartree	ΔG_{219K} / kJ mol^{-1}
***E*,*α*-1a 01**	−1095.732956	−1095.375206	25.52
***E*,*α*-1a 02**	−1095.729318	−1095.374566	27.20
***Z*,*α*-1a 01**	−1095.732570	−1095.375024	25.99
***Z*,*α*-1a 02**	−1095.731403	−1095.376128	23.10
***E*,*β*-1a 01**	−1095.741678	−1095.382975	5.12
***E*,*β*-1a 02**	−1095.737860	−1095.383558	3.59
***E*,*β*-1a 03**	−1095.738638	−1095.381663	8.56
***E*,*β*-1a 04**	−1095.738406	−1095.384270	1.72
***Z*,*β*-1a 01**	−1095.743360	−1095.384925	0.00
***Z*,*β*-1a 02**	−1095.740329	−1095.381090	10.07
***Z*,*β*-1a 03**	−1095.737793	−1095.378954	15.68
***Z*,*β*-1a 04**	−1095.738625	−1095.384032	2.35
***Z*,*β*-1a 05**	−1095.735536	−1095.380528	11.54
***Z*,*β*-1a 06**	−1095.735650	−1095.379767	13.54
***E*,*γ*-1a 01**	−1095.738216	−1095.380493	11.64
***E*,*γ*-1a 02**	−1095.741853	−1095.383440	3.90
***E*,*γ*-1a 03**	−1095.738483	−1095.383864	2.79
***E*,*γ*-1a 04**	−1095.736951	−1095.379236	14.94
***E*,*γ*-1a 05**	−1095.737038	−1095.382241	7.05
***Z*,*γ*-1a 01**	−1095.737413	−1095.379422	14.45
***Z*,*γ*-1a 02**	−1095.742396	−1095.383770	3.03
***Z*,*γ*-1a 03**	−1095.735844	−1095.380942	10.46
***Z*,*γ*-1a 04**	−1095.738633	−1095.384087	2.20
***Z*,*γ*-1a 05**	−1095.734835	−1095.379146	15.17
***E*,*δ*-1a 01**	−1095.732828	−1095.379232	14.95
***E*,*δ*-1a 02**	−1095.735612	−1095.377426	19.69

***E*,*δ*-1a 03**	−1095.738989	−1095.381294	9.53
***E*,*δ*-1a 04**	−1095.733276	−1095.379178	15.09
***E*,*δ*-1a 05**	−1095.735750	−1095.381609	8.71
***E*,*δ*-1a 06**	−1095.734896	−1095.378204	17.65
***E*,*δ*-1a 07**	−1095.733556	−1095.376910	21.04
***Z*,*δ*-1a 01**	−1095.738673	−1095.381377	9.31
***Z*,*δ*-1a 02**	−1095.737521	−1095.379167	15.12
***Z*,*δ*-1a 03**	−1095.735425	−1095.380871	10.64
***Z*,*δ*-1a 04**	−1095.730421	−1095.374776	26.65
***Z*,*δ*-1a 05**	−1095.736382	−1095.382182	7.20
***Z*,*δ*-1a 06**	−1095.735370	−1095.380924	10.50
***Z*,*δ*-1a 07**	−1095.731528	−1095.375207	25.51

Table S4. Summary of electronic energies, E_{SCF}, Gibbs energies, $G_{219\mathrm{K}}$, and relative Gibbs energies, $\Delta G_{219\mathrm{K}}$, for the isomers of **1b**. All values of $\Delta G_{219\mathrm{K}}$ are tabulated relative to the lowest-energy isomer of **1b**. All values are given as evaluated at the PBE0-D3/def2-SV(P) level of theory.

Isomer	E_{SCF} / Hartree	$G_{219\mathrm{K}}$ / Hartree	$\Delta G_{219\mathrm{K}}$ / kJ mol^{-1}
***E*,*α*-1b 01**	−1326.329764	−1325.913903	35.13
***E*,*α*-1b 02**	−1326.329254	−1325.912375	39.14
***E*,*α*-1b 03**	−1326.330206	−1325.913294	36.73
***E*,*α*-1b 04**	−1326.328153	−1325.914127	34.54
***Z*,*α*-1b 01**	−1326.330265	−1325.915297	31.47
***Z*,*α*-1b 02**	−1326.327412	−1325.912654	38.41
***Z*,*α*-1b 03**	−1326.330711	−1325.917191	26.50
***Z*,*α*-1b 04**	−1326.331095	−1325.916351	28.70
***Z*,*α*-1b 05**	−1326.329499	−1325.912456	38.93
***E*,*β*-1b 01**	−1326.336193	−1325.920135	18.77
***E*,*β*-1b 02**	−1326.341631	−1325.926446	2.20
***E*,*β*-1b 03**	−1326.334901	−1325.918072	24.18
***E*,*β*-1b 04**	−1326.337349	−1325.921220	15.92

***E*,*β*-1b 05**	−1326.339789	−1325.922722	11.98
***E*,*β*-1b 06**	−1326.340586	−1325.921688	14.69
***E*,*β*-1b 07**	−1326.339529	−1325.925193	5.49
***E*,*β*-1b 08**	−1326.341663	−1325.926761	1.37
***E*,*β*-1b 09**	−1326.337066	−1325.921894	14.15
***E*,*β*-1b 10**	−1326.337717	−1325.917963	24.47
***Z*,*β*-1b 01**	−1326.339963	−1325.924910	6.23
***Z*,*β*-1b 02**	−1326.339003	−1325.922341	12.98
***Z*,*β*-1b 03**	−1326.338920	−1325.926053	3.23
***Z*,*β*-1b 04**	−1326.341340	−1325.926574	1.86
***Z*,*β*-1b 05**	−1326.336576	−1325.922267	13.17
***Z*,*β*-1b 06**	−1326.336428	−1325.921766	14.49
***Z*,*β*-1b 07**	−1326.333161	−1325.920896	16.77
***Z*,*β*-1b 08**	−1326.338822	−1325.923744	9.29
***Z*,*β*-1b 09**	−1326.342696	−1325.926726	1.46
***E*,*γ*-1b 01**	−1326.334737	−1325.919762	19.75
***E*,*γ*-1b 02**	−1326.340859	−1325.925817	3.85
***E*,*γ*-1b 03**	−1326.335467	−1325.919398	20.70
***E*,*γ*-1b 04**	−1326.340177	−1325.922525	12.49
***E*,*γ*-1b 05**	−1326.341056	−1325.925377	5.01
***E*,*γ*-1b 06**	−1326.337167	−1325.921312	15.68
***E*,*γ*-1b 07**	−1326.339046	−1325.923956	8.74
***Z*,*γ*-1b 01**	−1326.337802	−1325.923089	11.01
***Z*,*γ*-1b 02**	−1326.336611	−1325.921922	14.08
***Z*,*γ*-1b 03**	−1326.339983	−1325.925903	3.62
***Z*,*γ*-1b 04**	−1326.336923	−1325.921956	13.99
***Z*,*γ*-1b 05**	−1326.336091	−1325.922845	11.65
***Z*,*γ*-1b 06**	−1326.342626	−1325.927283	0.00
***Z*,*γ*-1b 07**	−1326.340554	−1325.925204	5.46

***Z*,*γ*-1b 08**	−1326.339281	−1325.926646	1.67
***E*,*δ*-1b 01**	−1326.333932	−1325.920025	19.06
***E*,*δ*-1b 02**	−1326.331525	−1325.916638	27.95
***E*,*δ*-1b 03**	−1326.331585	−1325.917496	25.70
***E*,*δ*-1b 04**	−1326.333950	−1325.919739	19.81
***E*,*δ*-1b 05**	−1326.336964	−1325.921466	15.27
***E*,*δ*-1b 06**	−1326.336430	−1325.919396	20.71
***E*,*δ*-1b 07**	−1326.334695	−1325.918347	23.46
***E*,*δ*-1b 08**	−1326.336637	−1325.921647	14.80
***E*,*δ*-1b 09**	−1326.333283	−1325.919776	19.71
***E*,*δ*-1b 10**	−1326.335553	−1325.920723	17.23
***Z*,*δ*-1b 01**	−1326.336584	−1325.922131	13.53
***Z*,*δ*-1b 02**	−1326.335856	−1325.920566	17.64
***Z*,*δ*-1b 03**	−1326.337115	−1325.923018	11.20
***Z*,*δ*-1b 04**	−1326.333331	−1325.917587	25.46
***Z*,*δ*-1b 05**	−1326.336440	−1325.918593	22.82
***Z*,*δ*-1b 06**	−1326.334286	−1325.922014	13.83
***Z*,*δ*-1b 07**	−1326.337932	−1325.923306	10.44
***Z*,*δ*-1b 08**	−1326.335453	−1325.921372	15.52
***Z*,*δ*-1b 09**	−1326.336085	−1325.921737	14.56
***Z*,*δ*-1b 10**	−1326.333951	−1325.917563	25.52
***Z*,*δ*-1b 11**	−1326.335738	−1325.923665	9.50

7.3. NCI Plots

Qualitative analysis of noncovalent interactions was carried out by generating NCI plots. Based on the electron density and the derivative of its gradient, NCI plots visualize attractive, repulsive, and weak (van der Waals) interactions within a molecule or between two molecules.[21] At low densities in the reduced density gradient regions, weak interactions appear which are then mapped as isosurfaces over the molecule. The colour of the isosurface as blue, green, orange or red indicates strongly attractive, weakly attractive, weakly repulsive, and strongly repulsive interactions, respectively. A 2D plot of reduced density gradient *vs*. density and second Hessian λ ((λ2)ρ) also provides qualitative information about these interactions.

Single-point energy calculations were carried out at LC-PBE0/def2-TZVP level to generate wavefunction files using ORCA (v5.0.2).[20m-o] Multiwfn 3.8[S4] was used thereafter to generate cuboid and text files.

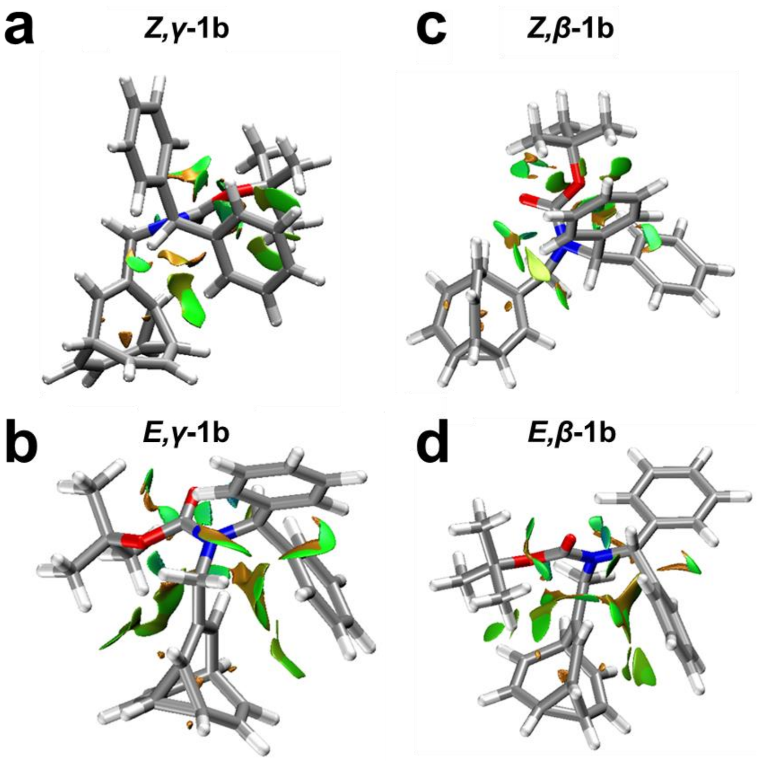

Figure S26. NCI analysis of (a) ***Z,γ*-1b** (b) ***Z,β*-1b** (c) ***E,γ*-1b** and (d) ***E,β*-1b** (grid size: 0.05 a.u; isosurface: 0.5; ρ range: -0.025 – 0.011 a.u) at the LC-PBE0/def2-TZVP//PBE0-D3/def-SV(P) level. The ***Z,γ*-1b** shows a strongly attractive interaction between the one Ph group and the bullvalene. The Ph-to-bullvalene interactions are less extensive for ***Z,β*-1b**. For ***E,γ*-1b** and ***E,β*-1b** the interactions cover a larger area, but include a substantial amount of repulsive character (in orange).

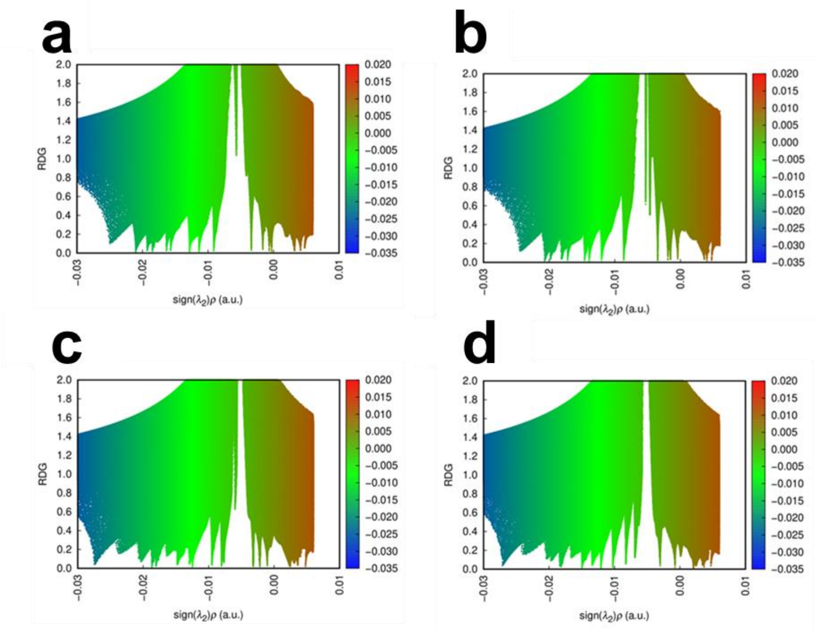

Figure S27. Plots of the reduced density gradient (RDG) versus the electron density multiplied by the sign of the second Hessian eigenvalue at the LC-PBE0/def2-TZVP//PBE0-D3/def-SV(P) level: (a) ***Z,γ*-1b** (b) ***Z,β*-1b** (c) ***E,γ*-1b** and (d) ***E,β*-1b**.

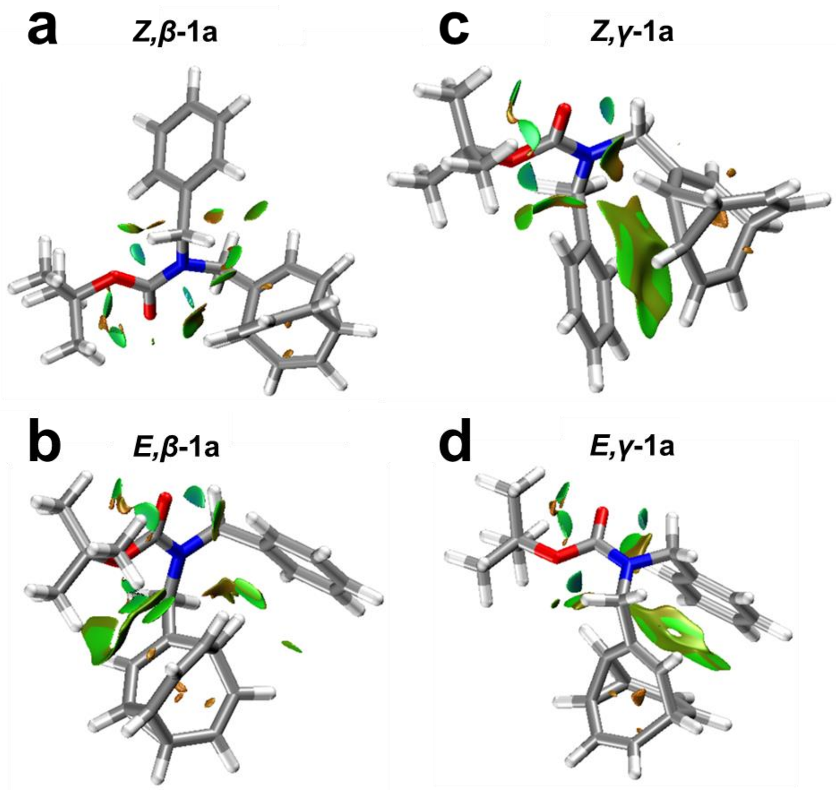

Figure S28. NCI analysis of (a) ***Z*,*β*-1a**, (b) ***E*,*β*-1a**, (c) ***Z*,*γ*-1a** and(d) ***E*,*γ*-1a** (grid size: 0.05 a.u; isosurface: 0.5; ρ range: -0.025 – 0.011 a.u) at the LC-PBE0/def2-TZVP//PBE0-D3/def-SV(P) level.

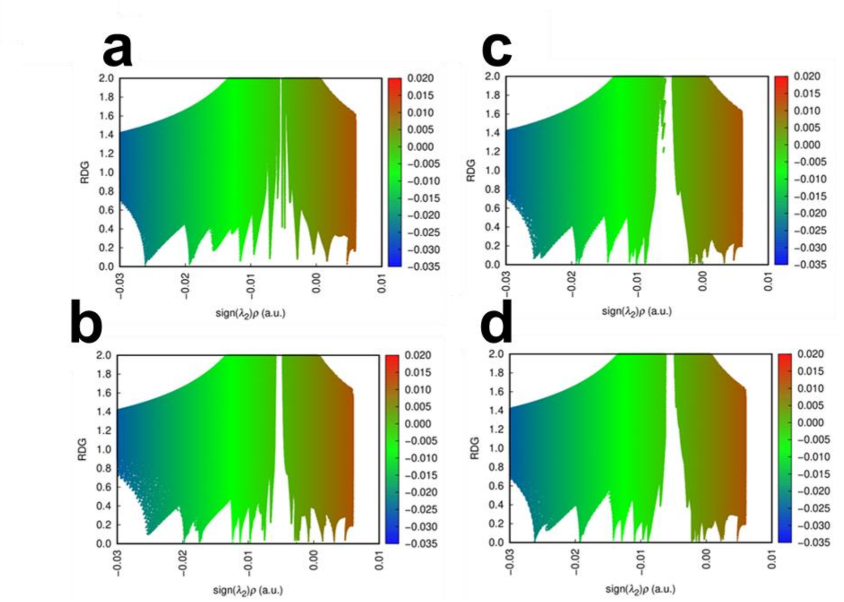

Figure S29. Plots of the reduced density gradient (RDG) versus the electron density multiplied by the sign of the second Hessian eigenvalue at the LC-PBE0/def2-TZVP//PBE0-D3/def-SV(P) level: (a) ***Z,β*-1a**, (b) ***E,β*-1a**, (c) ***Z,γ*-1a** and (d) ***E,γ*-1a**.

8. References

S1. O. V. Dolomanov, L. J. Bourhis, R. J. Gildea, J. A. K. Howard and H. Puschmann, *J. Appl. Crystallogr.* 2009, **42**, 339–341.

S2. G. M. Sheldrick, *Acta Crystallogr. A* 2008, **64**, 112–122.

S3. M. Yoshida, Y. Komatsuzaki and M. Ihara, *Org. Lett.* 2008, **10**, 2083–2086.

S4. T. Lu and F. Chen, *J. Comput. Chem.* 2012, **33**, 580–592.
